# MYC is a clinically significant driver of mTOR inhibitor resistance in breast cancer

**DOI:** 10.1084/jem.20211743

**Published:** 2023-08-29

**Authors:** Jinhyuk Bhin, Julia Yemelyanenko, Xue Chao, Sjoerd Klarenbeek, Mark Opdam, Yuval Malka, Liesbeth Hoekman, Dinja Kruger, Onno Bleijerveld, Chiara S. Brambillasca, Justin Sprengers, Bjørn Siteur, Stefano Annunziato, Matthijs J. van Haren, Nathaniel I. Martin, Marieke van de Ven, Dennis Peters, Reuven Agami, Sabine C. Linn, Epie Boven, Maarten Altelaar, Jos Jonkers, Daniel Zingg, Lodewyk F.A. Wessels

**Affiliations:** 1 Division of Molecular Carcinogenesis, Netherlands Cancer Institute, Amsterdam, Netherlands; 2 Division of Molecular Pathology, Netherlands Cancer Institute, Amsterdam, Netherlands; 3 Oncode Institute, Utrecht, Netherlands; 4 Department of Biomedical System Informatics, Gangnam Severance Hospital, Yonsei University College of Medicine, Seoul, Republic of Korea; 5 Experimental Animal Pathology, Netherlands Cancer Institute, Amsterdam, Netherlands; 6 Division of Oncogenomics, Netherlands Cancer Institute, Amsterdam, Netherlands; 7 Proteomics Facility, Netherlands Cancer Institute, Amsterdam, Netherlands; 8 Department of Medical Oncology, Amsterdam University Medical Center, Vrije Universiteit Amsterdam/Cancer Center Amsterdam, Amsterdam, Netherlands; 9 Mouse Clinic for Cancer and Aging, Netherlands Cancer Institute, Amsterdam, Netherlands; 10 Biological Chemistry Group, Institute of Biology Leiden, Leiden University, Leiden, Netherlands; 11 Core Facility Molecular Pathology and Biobanking, Netherlands Cancer Institute, Amsterdam, Netherlands; 12 Department of Pathology, Netherlands Cancer Institute, Amsterdam, Netherlands; 13 Biomolecular Mass Spectrometry and Proteomics, Bijvoet Center for Biomolecular Research, Utrecht Institute for Pharmaceutical Sciences, Utrecht University, Utrecht, Netherlands; 14 Netherlands Proteomics Centre, Utrecht, Netherlands

## Abstract

Targeting the PI3K–AKT–mTOR pathway is a promising therapeutic strategy for breast cancer treatment. However, low response rates and development of resistance to PI3K–AKT–mTOR inhibitors remain major clinical challenges. Here, we show that MYC activation drives resistance to mTOR inhibitors (mTORi) in breast cancer. Multiomic profiling of mouse invasive lobular carcinoma (ILC) tumors revealed recurrent *Myc* amplifications in tumors that acquired resistance to the mTORi AZD8055. MYC activation was associated with biological processes linked to mTORi response and counteracted mTORi-induced translation inhibition by promoting translation of ribosomal proteins. In vitro and in vivo induction of MYC conferred mTORi resistance in mouse and human breast cancer models. Conversely, AZD8055-resistant ILC cells depended on MYC, as demonstrated by the synergistic effects of mTORi and MYCi combination treatment. Notably, MYC status was significantly associated with poor response to everolimus therapy in metastatic breast cancer patients. Thus, MYC is a clinically relevant driver of mTORi resistance that may stratify breast cancer patients for mTOR-targeted therapies.

## Teaser

Bhin et al. show that MYC activation drives resistance to mTOR inhibitors and is significantly associated with poor response to mTOR inhibition in breast cancer patients, suggesting the potential of MYC as a biomarker for predicting resistance to mTOR-targeted therapies.

## Introduction

The PI3K–AKT–mTOR signaling pathway is central to multiple cellular processes and is frequently dysregulated in human cancer. In breast cancer, the PI3K–AKT–mTOR pathway is often activated by genomic abnormalities, most commonly by *PIK3CA* hotspot mutations, *PTEN* copy number loss, or activation of upstream signaling cues derived from receptor tyrosine kinases (Ciriello et al., 2015; Mukohara, 2015). High prevalence of PI3K–AKT–mTOR pathway activation in breast cancer has guided clinical trials evaluating small-molecule compounds targeting this pathway (Bahrami et al., 2018). This has resulted in the approval of two drugs for breast cancer, everolimus (Baselga et al., 2012) and alpelisib (André et al., 2019), targeting mTOR and PIK3CA, respectively.

Although PI3K–AKT–mTOR signaling blockade is a promising therapeutic strategy for breast cancer, predicting patient response using biomarkers has remained challenging, which has compromised the effectiveness of these targeted therapies (Lee et al., 2015; Yi and Ma, 2017). For the everolimus–exemestane combination treatment in estrogen receptor–positive (ER+) breast cancer, the clinical benefit rate, defined as the proportion of patients with a complete response (CR), partial response (PR), or stable disease (SD) for at least 16 wk, was about 51%, and the overall response rate (CR + PR) was about 16% (Baselga et al., 2012). This highlights the need for biomarkers to guide patient stratification for this treatment. Moreover, the acquisition of resistance to inhibitors targeting PI3K or mTOR is a major clinical obstacle, potentially induced by multiple types of resistance mechanisms (Brandão et al., 2019; Formisano et al., 2020). *PIK3CA* and *MTOR* gatekeeper and non-gatekeeper mutations can give rise to resistance (Zunder et al., 2008; Wu et al., 2015; Lorenz and Heitman, 1995; Nakanishi et al., 2016). Activation of independent proliferation pathways (Rozengurt et al., 2014; O’Reilly et al., 2006) and alterations of downstream targets, such as eIF4E and 4E-BP1 (Alain et al., 2012; Cope et al., 2014; Jastrzebski et al., 2018), may represent alternative strategies for cancers to acquire resistance to PI3K and mTOR inhibition. However, most of these findings have been identified in in vitro models and/or by the use of preclinical mTOR inhibitors (mTORi); thus the in vivo and clinical relevance of these findings for breast cancer has remained elusive.

Oncogenomic studies have demonstrated frequent activation of PI3K–AKT–mTOR signaling in the invasive lobular carcinoma (ILC) histological subtype of breast cancers, suggesting this pathway to be a particularly promising therapeutic target for ILC (Ciriello et al., 2015; Curtis et al., 2012; Michaut et al., 2016). Conceivably, we previously observed strong activation of and dependence on PI3K–AKT–mTOR signaling in the transplantable *K14-Cre*;*Cdh1*^F/F^;*Trp53*^F/F^ (KEP) mouse model of ILC (Klarenbeek et al., 2020; Doornebal et al., 2013), thus representing an optimal in vivo model system to study mechanisms of resistance during mTOR blockade. Using the transplantable KEP model, we here performed multiomic analyses of ILC tumors obtained during long-term mTORi treatment to study in vivo mechanisms of acquired resistance to mTOR inhibition in breast cancer. We uncovered that MYC activation is a hallmark of mTORi resistance, coordinating cell-intrinsic and -extrinsic processes involved in response to mTOR inhibition, including the compensation of suppressed protein translation by mTORi. Orthotopic modeling of mouse ILC tumors and human breast cancer–derived xenografts confirmed that MYC is an in vivo and clinically relevant driver of mTORi resistance. We also showed that MYC status is significantly associated with clinical response to the mTORi everolimus in breast cancer patients, thus establishing MYC as a clinically significant driver of mTORi resistance.

## Results

### Molecular profiling of AZD8055-resistant KEP tumors

**Figure 1.**
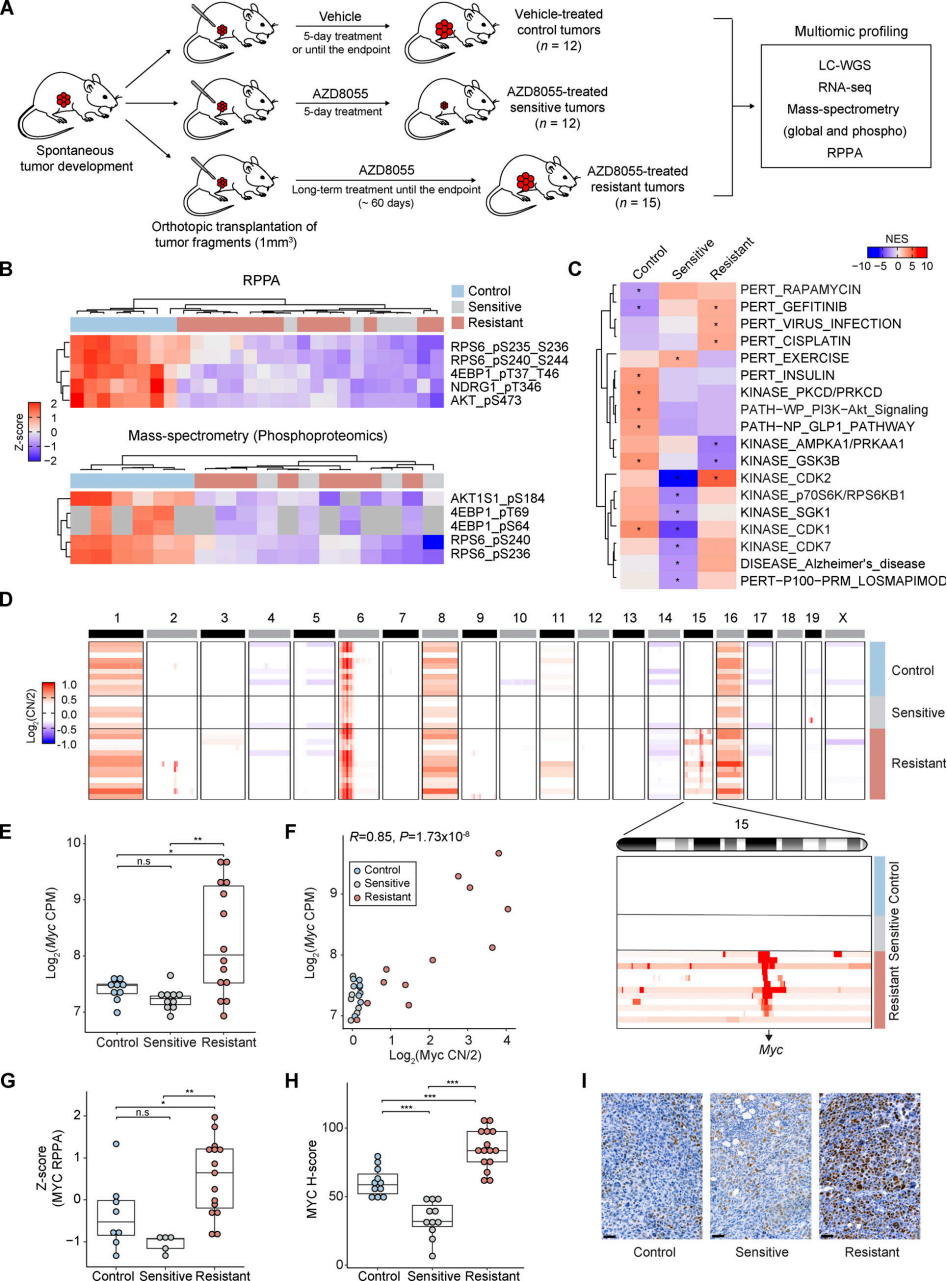
**Upregulation of MYC in AZD8055-resistant KEP tumors. (A)** Scheme representing the experimental setup to generate vehicle-treated control and AZD8055-treated sensitive and resistant KEP mammary tumors used for the multiomic data analyses. Endpoint was reached when mammary tumor volume was >1,500 mm^3^. **(B)** mTOR downstream phosphosites measured by RPPA (8 control, 5 sensitive, and 15 resistant KEP tumors) and MS-based phosphoproteomics (6 control, 4 sensitive, and 8 resistant KEP tumors). Intensities from each assay were standardized using Z-scores. Gray cells in MS data indicate missing values. **(C)** Phosphosite-specific signature analysis based on the PTMSigDB database (Krug et al., 2019). The average intensities for each KEP tumor group were used to identify representative signatures. *FDR < 0.05. **(D)** Copy number (CN) ratio (log_2_CN/2) of 10 control, 6 sensitive, and 13 resistant KEP tumors for all chromosomes and chromosome 15 (inset) highlighting focal MYC amplifications in resistant tumors. **(E)** *Myc* mRNA expression (log_2_CPM) across 9 control, 10 sensitive, and 14 resistant KEP tumors. **(F)** Pearson’s correlation analysis of *Myc* copy number ratio (log_2_CN/2) versus *Myc* mRNA expression (log_2_CPM). P value was calculated with two-tailed *t*-transformations of Pearson’s correlation coefficient. **(G)** Z-score standardized MYC protein levels across 8 control, 5 sensitive, and 15 resistant KEP tumors as measured by RPPA. **(H)** H-score quantifications of MYC staining intensities across 13 control, 11 sensitive, and 15 resistant tumors. **(I)** Representative MYC IHC stainings of control, sensitive, and resistant KEP tumors in H. Scale bars, 50 μm. In E–H, data are represented as median ± interquartile range (IQR; box) and quartiles ± 1.5 × IQR (whiskers), and one-way ANOVA and Tukey’s post-hoc test were performed to compute adjusted P values (*P < 0.05; **P < 0.01; ***P < 0.001; n.s, not significant). Source data are available for this figure: SourceData F1.

**Figure S1.**
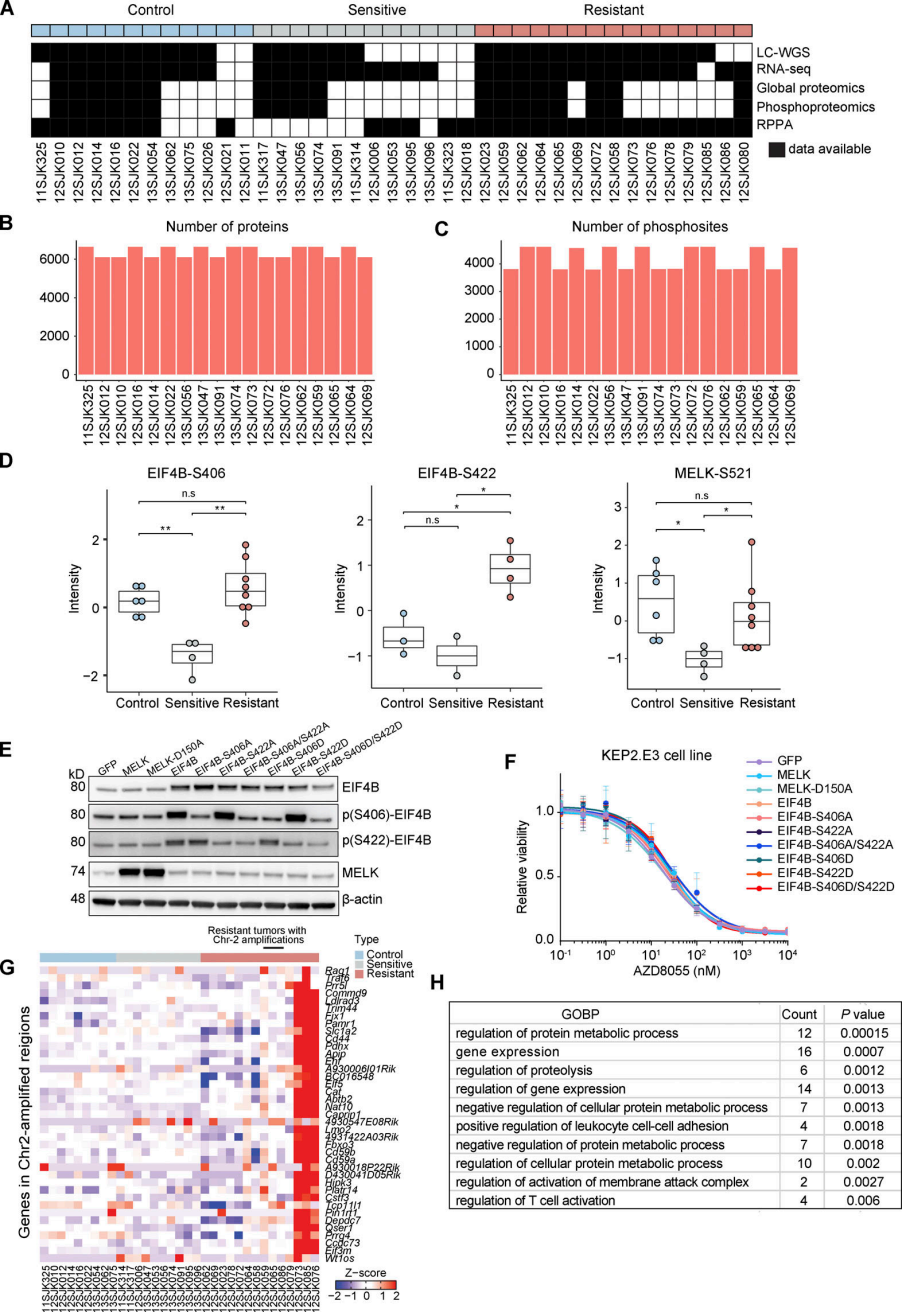
**Multiomic molecular profiling of KEP tumors. (A)** Control (*n* = 12), sensitive (*n* = 12), and resistant (*n* = 15) KEP tumors were analyzed by LC-WGS, RNA-seq, MS-based expression proteomics, phosphoproteomics, and RPPA. **(B)** The number of proteins quantified by MS-based expression proteomics. **(C)** The number of phosphosites quantified by MS-based phosphoproteomics. **(D)** Phosphorylation levels of EIF4B and MELK were measured by MS-based phosphoproteomics across six control, four sensitive, and eight resistant KEP tumors. Phosphosites for some tumors were not available due to the limited dynamic range of MS. One-way ANOVA and Tukey’s post-hoc tests were performed to compute adjusted P value (*P < 0.05; **P < 0.01; n.s, not significant). **(E)** Western blots for EIF4B, MELK, and their phosphosites on KEP2.E3 cells transduced with lentiviral overexpression vectors for *GFP*, *Eif4b*, its phosphomimetic (*S406D* and *S422D*) and non-phosphorylatable mutants (*S406A* and *S422A*), *Melk*, or its non-phosphorylatable mutant (*S150A*). Data represent one experiment of two independent experiments. **(F)** Dose–response curves of KEP2.E3 cells expressing indicated *Eif4b* or *Melk* variants, treated with AZD8055 for 3 d, and assayed using CellTiter-Blue reagent. Data are represented as mean ± standard deviation of five replicas per group of two independent experiments. **(G)** Expression of genes present in Chr-2 amplicon identified in several resistant KEP tumors. Log_2_(CPM) values were transformed to Z-scores. Data are shown for 9 control, 10 sensitive, and 14 resistant KEP tumors. **(H)** GOBPs significantly enriched for the Chr-2 amplicon genes.

We previously established an in vivo experimental setting to investigate mTORi resistance in the transplantable KEP mouse model of ILC (Klarenbeek et al., 2020; Doornebal et al., 2013; Derksen et al., 2006). In this setting, immunocompetent mice bearing orthotopically transplanted KEP tumors were enrolled into an intervention study using the mTORi AZD8055, and mammary tumor samples were harvested from vehicle-treated control tumors, sensitive tumors after 5 d of treatment, and resistant tumors that progressed under long-term treatment (Fig. 1 A). Tumors from the three cohorts were subjected to multiomic analyses, that is, low-coverage whole genome sequencing (LC-WGS), RNA sequencing (RNA-seq), reverse phase protein array (RPPA) profiling, mass spectrometry (MS)–based expression proteomics, and MS-based phosphoproteomics (Fig. S1 A). For MS, we quantified 7,003 proteins and 5,141 phosphosites on average using the Tandem Mass Tag (TMT) isobaric labeling approach (Fig. S1, B and C). Together, we established a high-quality multiomic data collection to assess genomic and (phospho)proteomic changes during mTORi sensitivity and resistance.

Consistent with previous RPPA analysis (Klarenbeek et al., 2020), MS-phosphoproteomic data corroborated sustained inhibition of mTOR downstream targets in the resistant tumors, indicating that the acquired resistance to AZD8055 was not driven by the recovery of mTOR activity (Fig. 1 B). Phosphosite-specific signature analysis using PTMSigDB (Krug et al., 2019) demonstrated that both sensitive and resistant tumors had low enrichment of the PI3K–AKT signature and high enrichment of the rapamycin signature as compared with control tumors, consistently supporting sustained suppression of the PI3K–AKT–mTOR signaling in the resistant tumors. Moreover, suppression of cell cycle kinase signatures (e.g., CDK1, CDK2, and CDK7) in sensitive tumors and re-enrichment of these signatures in resistant tumors indicated that resistant tumors were actively proliferating (Fig. 1 C). The only mTOR downstream target that regained phosphorylation in resistant tumors was EIF4B (Fig. S1 D). However, overexpression of EIF4B and its phospho-mimicking variants (S406D, S422D) in the KEP tumor–derived cell line KEP2.E3 (Klarenbeek et al., 2020) did not confer resistance to AZD8055 in vitro (Fig. S1, E and F). Likewise, overexpression of MELK, which can act as an alternative upstream activator to EIF4B (Wang et al., 2016) and which was reactivated in resistant tumors as compared with sensitive tumors, had no effect on AZD8055 sensitivity in KEP2.E3 (Fig. S1, D–F). This suggested that recovery of EIF4B and MELK phosphorylation represented a marker of proliferative tumor progression rather than a driver of resistance to AZD8055.

### *Myc* is recurrently amplified in AZD8055-resistant KEP tumors

Next, we analyzed genome-wide copy number profiles of control, sensitive, and resistant KEP tumors using LC-WGS. Interestingly, *Myc* was focally amplified in the majority of the resistant tumors (10/13), while control and sensitive tumors harbored normal *Myc* copy numbers (Fig. 1 D). The three remaining resistant tumors contained a focal amplification of chromosome 2 (Chr-2; 101,638,292–105,126,510; Fig. 1 D). The amplified region covered 35 protein-coding genes including translation initiation factors *Eif3m* and *Eif5* that are involved in processes such as cell adhesion, proteolysis, and immune responses (Fig. 1 D and Fig. S1, G and H). RNA-seq revealed that *Myc* expression was significantly upregulated in the resistant tumors as compared with control and sensitive tumors (Fig. 1 E), and *Myc* copy number amplification was highly correlated with *Myc* upregulation (Fig. 1 F). We next assessed the protein level of MYC using RPPA data, demonstrating a higher abundance of MYC in the resistant tumors as compared with control and sensitive tumors (Fig. 1 G). Finally, we quantified *Myc *expression in tumor cells using immunohistochemistry (IHC), which consistently revealed MYC upregulation in tumor cells from resistant samples versus tumor cells from control or sensitive samples (Fig. 1, H and I). Together, these data demonstrated that prolonged inhibition of mTOR fosters the genomic evolution of tumor cells resulting in the amplification and upregulation of MYC.

### MYC governs the transcriptional landscape of AZD8055-resistant tumors

**Figure 2.**
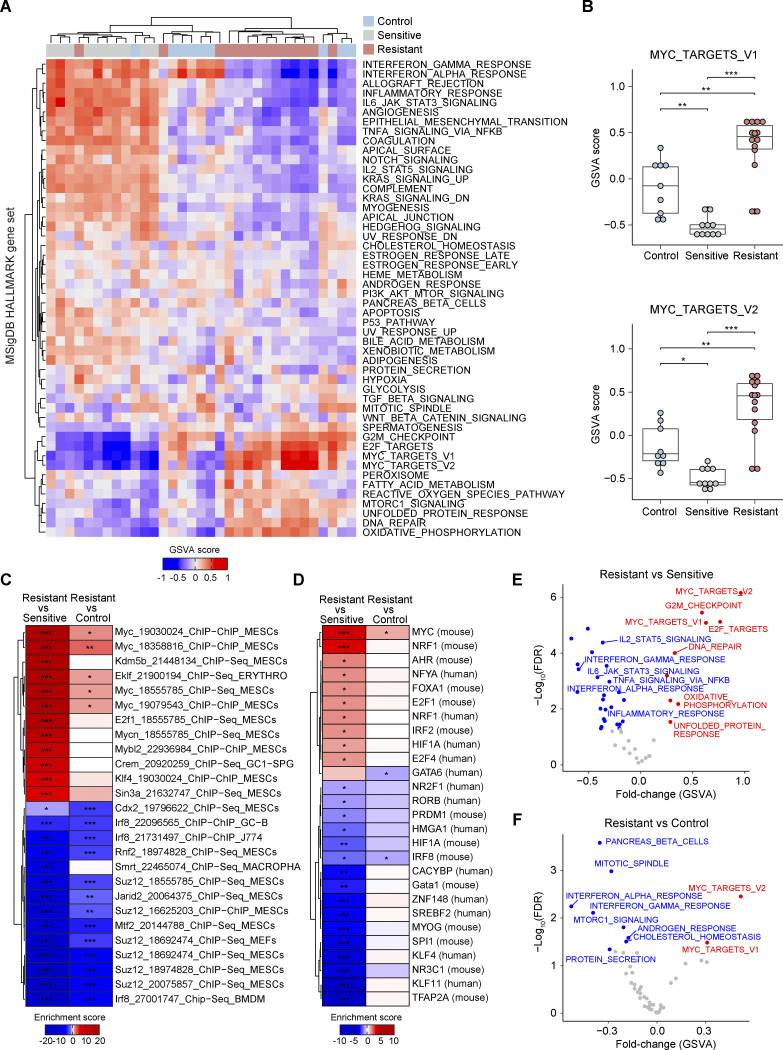
**Functional MYC activity in AZD8055-resistant tumors. (A)** GSVA analysis for 50 MSigDB Hallmark gene sets across RNA-seq profiles derived from 9 control, 10 sensitive, and 14 resistant KEP tumors. Hierarchical clustering (Euclidean distance and complete-linkage clustering) was performed for GSVA scores. **(B)** GSVA scores for the two MSigDB Hallmark MYC target gene sets. Data are represented as median ± IQR (box) and quartiles ± 1.5 × IQR (whiskers), and one-way ANOVA and Tukey’s post-hoc test were performed to compute adjusted P values (*P < 0.05; **P < 0.01; ***P < 0.001). **(C and D)** TF target and motif enrichment analyses for upregulated and downregulated genes in resistant vs. sensitive and resistant vs. control comparisons (RNA-seq profiles derived from 9 control, 10 sensitive, and 14 resistant KEP tumors). TF targets and motifs were obtained from ChEA2016 (C) and TRANSFAC_and_JASPAR_PWMs (D), all collected in the EnrichR database (Kuleshov et al., 2016). TF enrichment scores were defined as −log_10_(FDR) for upregulated genes in each comparison and as the additive inverse of −log_10_(FDR) for downregulated genes in each comparison and computed by Fisher’s exact test followed by Benjamini & Hochberg correction. Row labels in C, “TF name”_“Pubmed ID”_“experimental assay”_“cell line”. *FDR < 0.05; **FDR < 0.01; ***FDR < 0.001. **(E and F)** GSVA analysis for resistant versus sensitive (E) and resistant versus control (F) tumors using MS-based expression proteomic data (6 control, 4 sensitive, and 8 resistant KEP tumors). GSVA scores were compared using Student’s *t* test. Red and blue dots, significantly (FDR < 0.05) up- and downregulated in resistant tumors.

**Figure S2.**
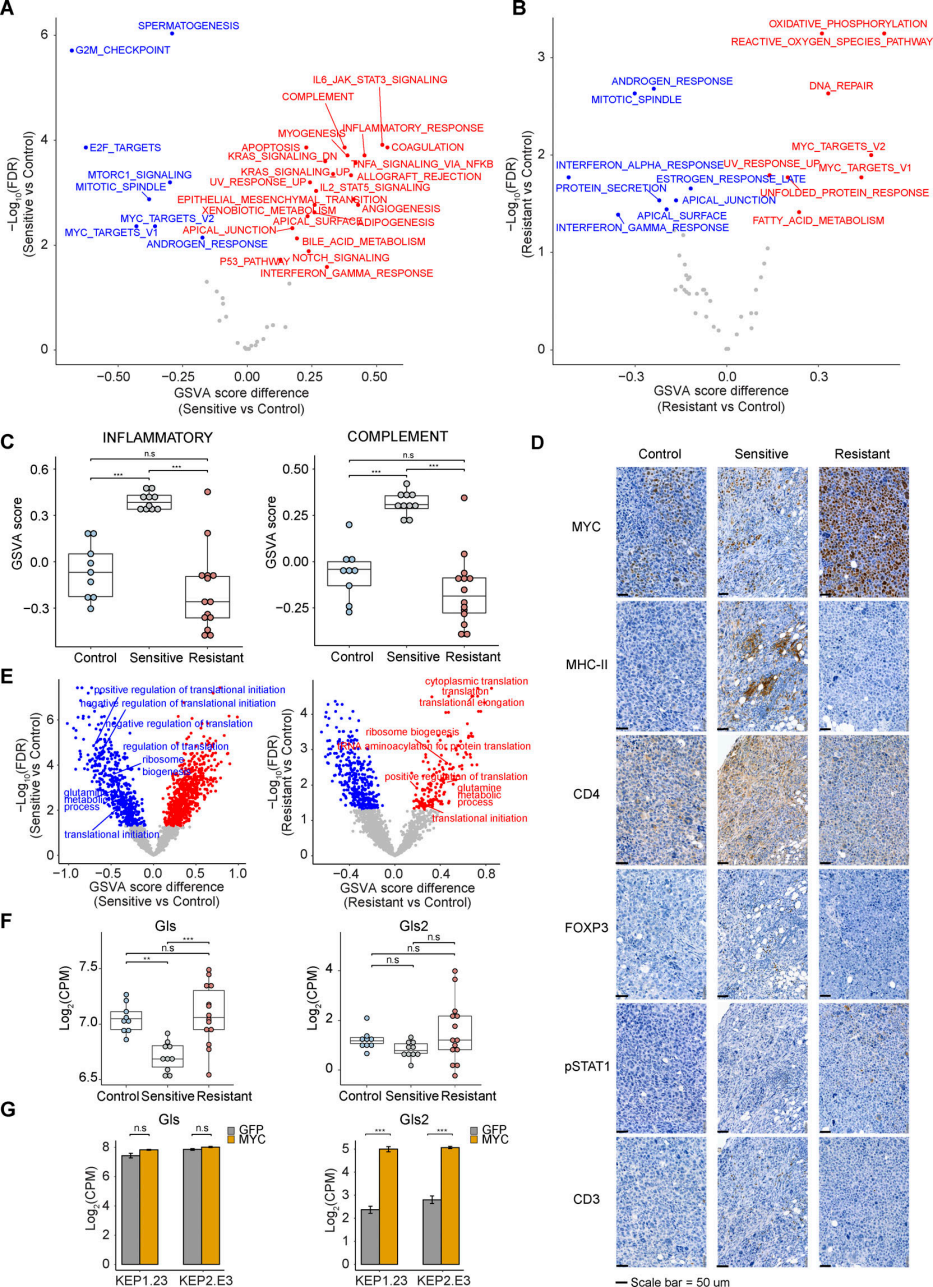
**Biological processes differentially regulated in different KEP tumor groups. (A and B)** GSVA analysis for sensitive versus control (A) and resistant versus control (B) tumors using RNA-seq data. GSVA scores were compared using Student’s *t* test. Red and blue dots, significantly (FDR < 0.05) up- and downregulated in resistant tumors. **(C)** GSVA scores for immune-related gene sets (INFLAMMATORY, COMPLEMENT) across 9 control, 10 sensitive, and 14 resistant KEP tumors. Data are represented as median ± IQR (box) and quartiles ± 1.5 × IQR (whiskers), and one-way ANOVA and Tukey’s post-hoc test were performed to compute adjusted P values. **(D)** Representative IHC staining of MYC and diverse immune cell markers across different KEP tumor groups. Scale bars, 50 μm. **(E)** GSVA analysis for sensitive versus control tumors (left) and resistant versus control tumors (right) using RNA-seq data based on GOBP gene sets. GSVA scores were compared using the Student’s *t* test. Red and blue dots, significantly (FDR < 0.05) up- and downregulated in sensitive tumors (left) and resistant tumors (right). Translation- and glutamine metabolism–associated gene sets are shown in the graph. **(F and G)** Expression (log_2_(CPM)) of glutaminolysis enzymes *Gls* and *Gls2* across different KEP tumor groups (*n* = 9, 10, and 14 for control, sensitive, and resistant tumors, respectively; F) and KEP cell lines expressing *GFP* or *Myc* (two replica per group; G). In C and F, data are represented as median ± IQR (box) and quartiles ± 1.5 × IQR (whiskers), and one-way ANOVA and Tukey’s post-hoc test were performed to compute adjusted P values (**P < 0.01; ***P < 0.001; n.s, not significant).

To explore the biological processes underlying resistance to prolonged mTOR inhibition and MYC upregulation, we performed gene set variation analysis (GSVA; Hänzelmann et al., 2013) for 50 MSigDB hallmark gene sets (Liberzon et al., 2015) using the RNA-seq profiles (Fig. 2 A). GSVA displayed the estimated activities of the gene sets across the tumor groups (Fig. S2, A and B). Compared with control and sensitive tumors, MYC_TARGETS_V1 and V2 displayed significantly higher scores in the resistant tumors (Fig. 2 B). This indicated functional activation of MYC in AZD8055-resistant tumors. We next performed transcription factor (TF) target enrichment analysis using the TF target information collected from previously published genome-wide chromatin immunoprecipitation (ChIP) assays and motif-based prediction, as deposited in the EnrichR resource (Kuleshov et al., 2016). Upregulated genes in resistant tumors showed the strongest enrichment for genes containing binding sites for MYC as well as MYC-binding motifs (Fig. 2, C and D). Finally, MS-expression proteomics data similarly revealed the activation of MYC targets in resistant tumors as compared with control and sensitive tumors (Fig. 2, E and F). Taken together, these data show that during long-term AZD8055 treatment, MYC governs a transcriptional program potentially driving resistance to mTORi.

### MYC directs biological processes associated with mTORi response

**Figure 3.**
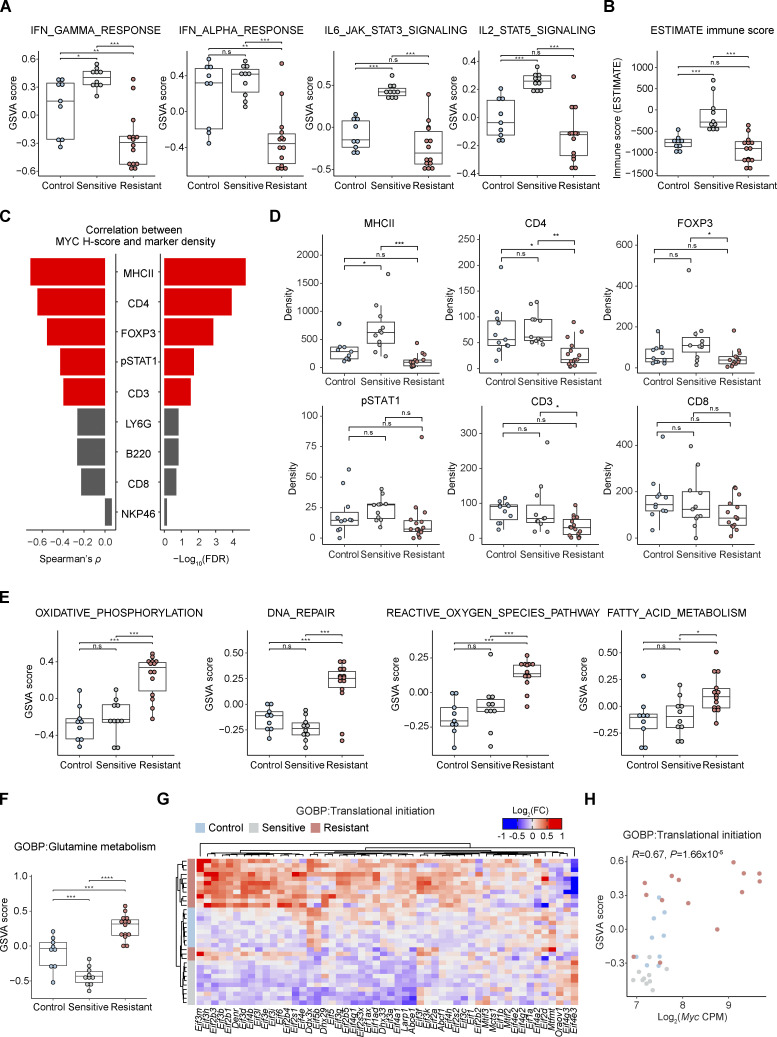
**Biological processes associated with AZD8055 efficacy. (A)** GSVA scores for immune-related MSigDB Hallmark gene sets using KEP tumor RNA-seq profiles. **(B)** Immune infiltration scores based on RNA-seq data inferred by ESTIMATE (Yoshihara et al., 2013). **(C)** Correlations between MYC protein H-scores and immune cell densities based on quantified IHC for MYC and indicated immune markers. Spearman’s ρ and −log_10_(FDR) are depicted as bar plots and red bars indicate significant correlations (FDR < 0.05). P values were calculated with two-tailed *t*-transformations of Spearman’s correlation coefficient. **(D)** Immune cell densities based on quantified IHC for indicated immune markers. **(E and F)** GSVA scores for MSigDB Hallmark gene sets (E) and GO glutamine metabolism (F) upregulated in resistant tumors. **(G)** Heatmap displaying mRNA expression of translation initiation genes obtained from the GO Biological Processes (GOBP) translational initiation gene set. Hierarchical clustering (Euclidean distance and complete-linkage clustering) was performed on log_2_-fold-change (log_2_FC) of CPM values centered on the median expression of control tumors. **(H)** Pearson’s correlation analysis of *Myc* mRNA expression (log_2_CPM) vs. GOBP translation initiation gene set scores. P value was calculated with two-tailed *t*-transformations of Pearson’s correlation coefficient. In A, B, and E–H, 9 control, 10 sensitive, and 14 resistant KEP tumors, and in C and D, 13 control, 11 sensitive, and 15 resistant KEP tumors were analyzed. In A, B, and D–F, data are represented as median ± IQR (box) and quartiles ± 1.5 × IQR (whiskers), and one-way ANOVA and Tukey’s post-hoc tests were performed to compute adjusted P values (*P < 0.05; **P < 0.01; ***P < 0.001; n.s, not significant).

To gain insight into the resistance-associated transcriptional programs mediated by MYC, we further explored the GSVA results (Fig. 2 A). Apart from terms directly connected to MYC activity, we also observed dynamic changes of immune-related pathways in the process of acquiring AZD8055 resistance (Fig. S2, A and B). The interferon α/γ response pathways were significantly downregulated in resistant tumors (Fig. 3 A), indicating a potential role of MYC in suppressing interferon signaling (Muthalagu et al., 2020; Swaminathan et al., 2020; Kim et al., 2016; Zimmerli et al., 2022). TF target enrichment analysis demonstrated that downregulated genes in resistant tumors were significantly enriched by the targets of immune-associated transcription regulators such as IRF8 and SMRT, supporting the suppression of immune response in resistant tumors (Fig. 2, C and D). Likewise, other immune processes such as IL6–JAK–STAT3 signaling, IL2–STAT5 signaling, inflammatory response, and complement were similarly triggered by short-term treatment of AZD8055 but became suppressed during resistance acquisition (Fig. 3 A and Fig. S2 C). Moreover, immune infiltration as assessed by Estimation of Stromal and Immune cells in Malignant Tumours using Expression data (ESTIMATE; Yoshihara et al., 2013) was predicted to be enriched in sensitive tumors, while resistant tumors showed an immune exclusion phenotype (Fig. 3 B). We experimentally validated the relationship between MYC and immune cell infiltration using IHC. Quantification of MYC intensities in tumor cells and densities of immune cell types in consecutive slides revealed a significant negative correlation between MYC expression and infiltration of CD3^+^, CD4^+^, FOXP3^+^, pSTAT1^+^, and MHCII^+^ immune cells (Fig. 3 C). These immune cell types were particularly enriched in tumors sensitive to AZD8055 but became excluded in AZD8055-resistant tumors (Fig. 3 D and Fig. S2 D). Our results suggested that MYC-driven immune exclusion contributes to AZD8055 resistance, in line with the previous finding that maintenance of AZD8055 sensitivity in KEP tumors partly depends on the adaptive immune system (Klarenbeek et al., 2020).

Besides immune-related processes, GSVA also revealed oxidative phosphorylation, the reactive oxygen species pathway, DNA repair, and fatty acid metabolism to be specifically upregulated in resistant tumors (Fig. 3 E). GSVA using Gene Ontology (GO) gene sets also showed upregulation of glutamine metabolism in resistant tumors (Fig. 3 F and Fig. S2 E). Enhanced mitochondrial energy metabolism and glutaminolysis were previously connected to mTORi resistance (Momcilovic et al., 2018; Tanaka et al., 2015). The glutaminolysis rate-limiting enzymes *Gls* and *Gls2* were upregulated in the resistant tumors, indicating enhanced glutamine-fueled oxidative phosphorylation in resistant tumors (Fig. S2 F). MYC can promote glutamine metabolism through transcriptional regulation of genes involved in glutaminolysis and glutamine uptake (Wise et al., 2008; Bott et al., 2015). Especially, *Gls2* expression was significantly correlated with *Myc* expression (P = 7.6 × 10^−3^) in resistant tumors and *Gls2* was upregulated in KEP cell lines upon *Myc *overexpression, suggesting that MYC stimulated the expression of *Gls2* (Fig. S2 G).

MYC can also act as a master regulator of translation by directly regulating the transcription of the genes involved in translation initiation and elongation, transfer RNA (tRNA) synthetases, and ribosomal proteins (van Riggelen et al., 2010; Schmidt, 2004). GSVA using GO gene sets demonstrated the upregulation of genes involved in protein translation initiation and elongation as well as ribosome biogenesis in resistant tumors, and the scores of these terms significantly correlated with *Myc* expression (Fig. 3, G and H; and Fig. S2 E). Intriguingly, gene sets connected to protein translation initiation were strongly diminished in AZD8055-sensitive tumors over controls (Fig. S2 E). Thus, MYC appeared to foster AZD8055 resistance by counteracting mTORi-mediated translation inhibition. Taken together, MYC regulated a range of tumor cell–intrinsic and –extrinsic biological processes associated with mTORi response and thus governed mTORi resistance.

### MYC confers resistance to mTOR blockade in vitro

**Figure 4.**
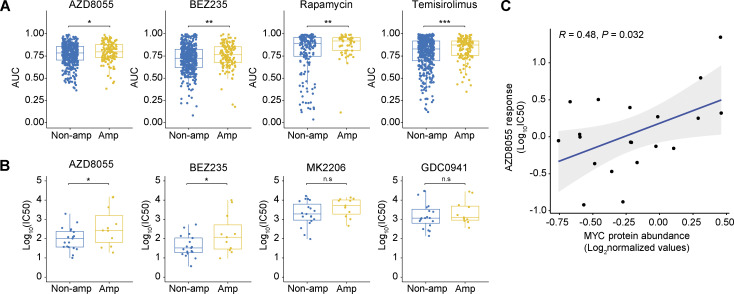
**Association between *MYC* status and mTORi response in human cancer cell lines. (A and B)** Comparisons of mTORi responses of cancer cell lines with and without *MYC* amplifications. In A, AUC values and *MYC* amplification status were obtained from the cBioPortal database (Gao et al., 2013). A total of 1,010 cell lines were analyzed (*MYC* amplification, *n* = 209; *MYC* non-amplified, *n* = 801). In B, IC50 values and *MYC* amplification status were obtained from a previously published breast cancer cell line dataset (Jastrzebski et al., 2018). Data are represented as median ± IQR (box) and quartiles ± 1.5 × IQR (whiskers) and P values were computed with one-tailed Student’s *t* tests (*P < 0.05; **P < 0.01; ***P < 0.001; n.s, not significant; *P* = 0.04, 0.04, 0.06, and 0.25 for AZD8055, BEZ235, MK2206, and GDC0941, respectively). A total of 30 cell lines were analyzed (*MYC* amplification, *n* = 11; *MYC* non-amplified, *n* = 19). **(C)** Correlation between AZD8055 response and MYC protein abundance in 20 human breast cancer cell lines. P value was calculated with two-tailed *t*-transformation of Pearson’s correlation coefficient. IC50 values were obtained from the GDSC database (Yang et al., 2013) and MYC protein levels, as measured by MS, were obtained from the CCLE database (Barretina et al., 2012). Source data are available for this figure: SourceData F4.

**Figure S3.**
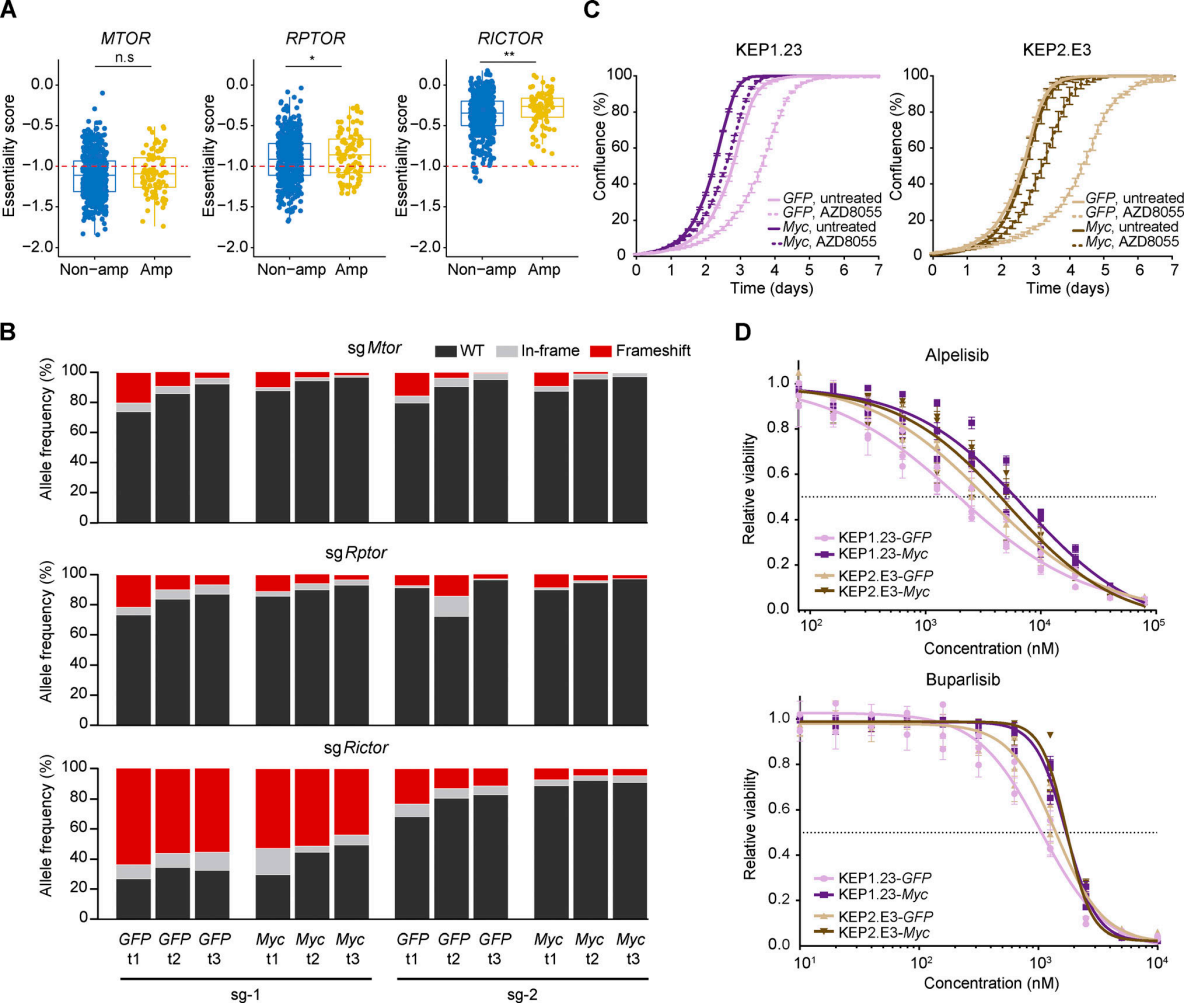
**Associations of MYC status versus response to PI3K/mTOR inhibition. (A)** Comparison of *MTOR*, *RPTOR*, and *RICTOR* gene essentiality scores of cancer cell lines with and without *MYC* amplifications. Scores and copy number data were obtained from the DepMap (CRISPR, Public 22Q1) and the Cell Model Passports (Tsherniak et al., 2017; van der Meer et al., 2019) database, respectively. Red dotted line (−1) indicates the average gene essentiality scores of known essential genes. Data are represented as median ± IQR (box) and quartiles ± 1.5 × IQR (whiskers) and P values were computed with one-tailed Student’s *t* tests (*P < 0.05; **P < 0.01; n.s, not significant). **(B)** KEP1.23 cells overexpressing *GFP* or *Myc* were transfected with lentiCRISPRv2-sgRNA-Cas9-Blast vectors containing the indicated sgRNAs targeting *Mtor*, *Rptor*, or *Rictor* and selected with blasticidin. At days 2 (t1), 4 (t2), and 6 (t3) after blasticidin selection, cells were lysed and allele frequencies for the indicated genes were determined using TIDE (Brinkman et al., 2014). **(C)** Incucyte Live-Cell proliferation assay of KEP cell lines overexpressing *GFP* or *Myc* and treated with vehicle or 25 nM AZD8055 for 7 d. Data are represented as mean ± SEM of three replica per group of two independent experiments. **(D)** Dose–response curves of KEP cell lines overexpressing *GFP* or *MYC*, treated with alpelisib or buparlisib for 3 d, and measured by SRB colorimetric assay. Data are represented as mean ± standard deviation of five technical replicas per group of three independent experiments.

To assess whether MYC functioned as a cell-intrinsic mediator of mTORi resistance, we leveraged pharmacogenomic datasets derived from a compendium of human cancer cell lines (Gao et al., 2013; Barretina et al., 2012; Yang et al., 2013). We evaluated the correlation between *MYC* copy number status and mTORi response. Cancer cell lines harboring *MYC* amplification versus lines with normal *MYC* copy numbers displayed significantly elevated area under the dose–response curve (AUC) values for multiple mTORi (Fig. 4 A). An independent dataset derived from 30 human breast cancer cell lines (Jastrzebski et al., 2018) validated the negative association between *MYC* amplification and mTORi response (Fig. 4 B). Of note, MYC protein levels and AZD8055 response values also significantly correlated across human breast cancer cell lines (Fig. 4 C). We examined whether, depending on their MYC status, cells would also differentially respond to genetic disruption of the mTOR complex. Therefore, we made use of the Cancer Dependency Map (Tsherniak et al., 2017) by focusing on the genes encoding the mTOR components MTOR, RPTOR, and RICTOR. This revealed that *MTOR*, and to a lesser extent *RPTOR* and *RICTOR*, are indispensable for the survival of the majority of cancer cell lines, regardless of *MYC* amplification status (Fig. S3 A). We functionally evaluated the essentiality of mTOR complex components in the mouse KEP tumor–derived KEP1.23 and KEP2.E3 cell lines (Klarenbeek et al., 2020) overexpressing either *GFP* or murine *Myc*. CRISPR/Cas9-based editing of *Mtor*, *Rptor*, or *Rictor* followed by Tracking of Indels by Decomposition (TIDE) analysis (Brinkman et al., 2014) revealed a continuous loss of frame-shift indels independent of *Myc* overexpression (Fig. S3 B). Thus, despite *Myc* amplification or experimental overexpression, mTOR components remained essential for cell survival, yet tolerance to mTORi appeared to be elevated.

**Figure 5.**
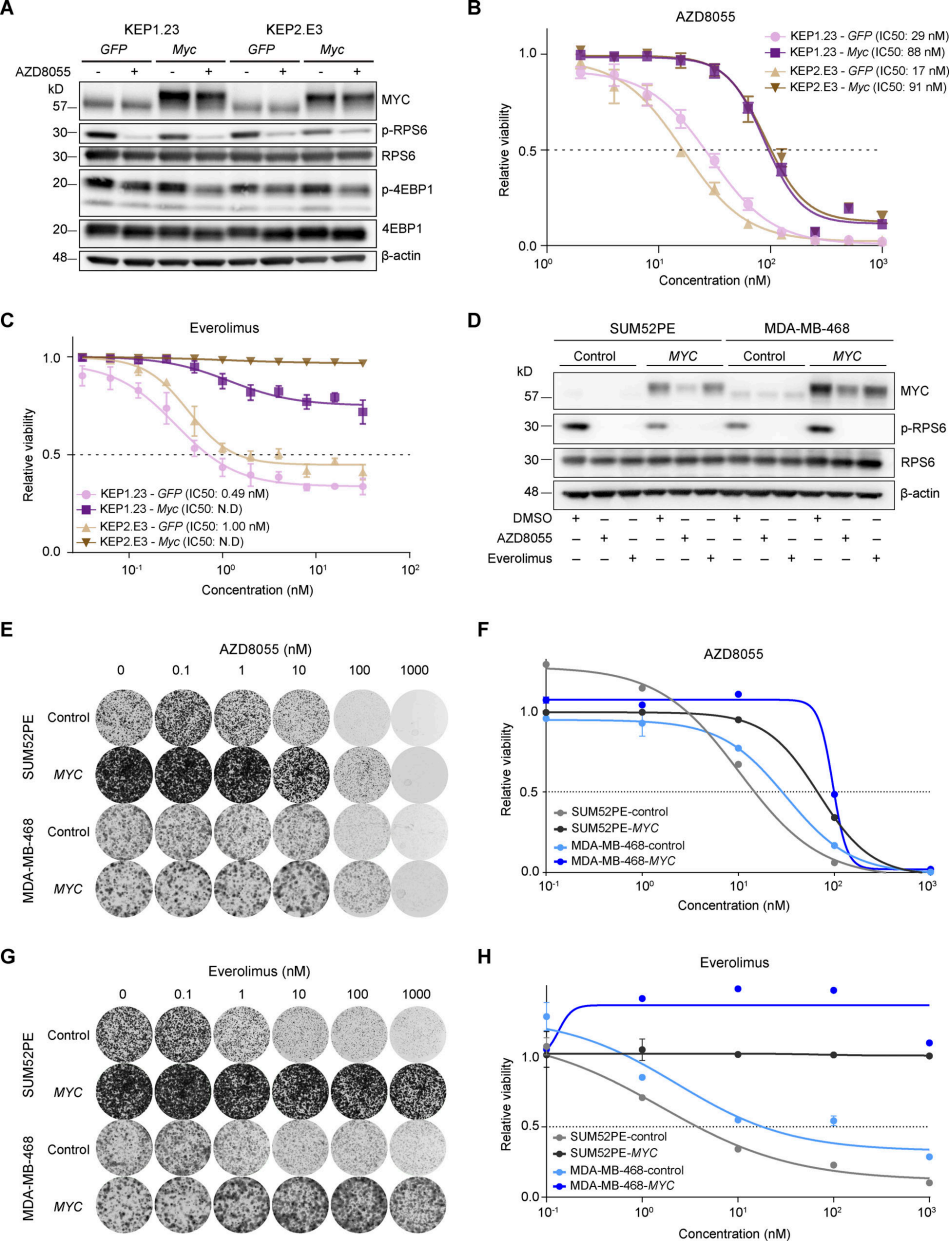
**MYC drives AZD8055 resistance in vitro. (A)** Western blots for MYC and mTOR signaling markers on KEP1.23 and KEP2.E3 cell lines transduced with *GFP* or *Myc*-overexpressing lentiviral vectors and treated for 24 h with vehicle or 25 nM AZD8055. Data represent one experiment. **(B and C)** Dose–response curves of KEP cell lines expressing *GFP* or *Myc*, treated with AZD8055 (B) or everolimus (C) for 3 d and measured by SRB colorimetric assay. Data are represented as mean ± standard deviation of five replicas per group of one representative experiment of *n* ≥ 3 independent experiments. N.D, not determined. **(D)** Western blots for MYC and (p-)RPS6 on serum-starved SUM52PE and MDA-MB-468 cell lines transduced with control or *MYC*-overexpressing lentiviral vectors and treated for 6 h with either vehicle DMSO, 200 nM AZD8055, or 10 nM everolimus. Data represent one experiment of two independent experiments. **(E–H)** Representative images (E and G) and corresponding quantification using CellTiter-Blue reagent (F and H) of long-term colony formation assays with SUM52PE and MDA-MB-468 cells transduced with control or *MYC*-overexpressing lentiviral vectors and treated with AZD8055 (E and F) or everolimus (G and H). Cells were treated with indicated drug doses for 10 d, and values were normalized to untreated conditions within each cell line. Data in F and H are represented as mean ± standard deviation of three technical replica per group across one representative experiment of two independent experiments. Source data are available for this figure: SourceData F5.

**Figure S4.**
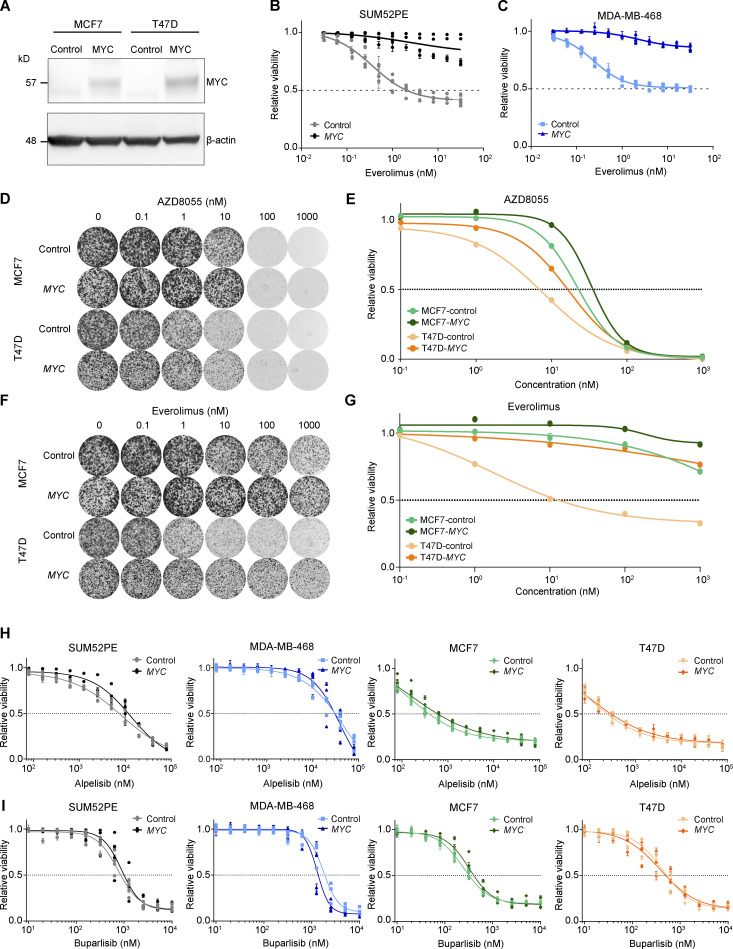
**In vitro drug response assay for inhibitors of PI3K and mTOR in human breast cancer cell lines. (A)** Western blots for MYC and β-actin control on MCF7 and T47D cell lines transduced with control or *MYC*-overexpressing lentiviral vectors. Data represent one experiment. **(B and C)** Dose–response curves of SUM52PE (A) and MDA-MB-468 (B) cell lines transduced with control or *MYC*-overexpressing lentiviral vectors and treated with everolimus for 3 d. Cell viability was measured by SRB colorimetric assay. Data are represented as mean ± standard deviation of five technical replicas per group of *n* ≥ 3 independent experiments. **(D–G)** Representative images (D and F) and corresponding quantification using CellTiter-Blue reagent (E and G) of long-term colony formation assays with MCF7 and T47D cells transduced with control or *MYC*-overexpressing lentiviral vectors and treated with AZD8055 (D and E) or everolimus (F and G). Cells were treated with indicated drug doses for 10 d, and values were normalized to untreated conditions within each cell line. Data in E and G are represented as mean ± standard deviation of three technical replicas per group of one representative experiment of two independent experiments. **(H and I)** Dose–response curves of T47D, MCF7, SUM52PE and MDA-MB-468 cell lines transduced with control or *MYC*-overexpressing lentiviral vectors, treated with alpelisib (H) or buparlisib (I) for 3 d, and measured by SRB colorimetric assay. Data are represented as mean ± standard deviation of five technical replica per group of *n* ≥ 3 independent experiments.

To functionally test whether MYC reduced the sensitivity to mTORi, we subjected *Myc*-overexpressing KEP cell lines and the corresponding *GFP*-expressing controls to AZD8055, which inhibited mTOR signaling regardless of *M*yc overexpression (Fig. 5 A). Importantly, drug–response assays showed a three to fivefold increased AZD8055 half-maximal inhibitory concentration (IC50) for KEP cells overexpressing *Myc* versus control cells (Fig. 5 B). Proliferation assays showed that mTOR inhibition efficiently hindered growth of control KEP cells, whereas *Myc*-overexpressing cells were less affected by AZD8055 (Fig. S3 C). Next, we subjected KEP cell lines to drug response assays using the clinically approved mTORi everolimus and the PI3K inhibitors (PI3Ki) alpelisib and buparlisib. Intriguingly, our results showed that MYC strongly induces resistance to everolimus, whereas the responses to alpelisib and buparlisib were only marginally affected by *Myc* overexpression (Fig. 5 C and Fig. S3 D). Finally, we overexpressed human *MYC* in the human breast cancer cell lines SUM52PE, MDA-MB-468, MCF7, and T47D (Fig. 5 D and Fig. S4 A). The mTORi AZD8055 and everolimus markedly suppressed mTOR signaling independently of MYC (Fig. 5 D). Consistent with the observations in KEP cell lines, colony formation and drug–response assays using SUM52PE and MDA-MB-468 demonstrated that *MYC* overexpression strongly reduced sensitivities to AZD8055 and everolimus (Fig. 5, E–H; and Fig. S4, B and C). In MCF7 and T47D cells, MYC also reduced mTORi sensitivity but to a lesser extent than in SUM52PE and MDA-MB-468 (Fig. S4, D–G). Noteworthy, in none of the four cell lines *MYC* overexpression affected sensitivity to the PI3Ki alpelisib and buparlisib (Fig. S4, H and I). Taken together, our results showed that MYC drives mTORi resistance in cell culture models of mouse and human breast cancer.

### MYC confers resistance to mTOR blockade in vivo

**Figure 6.**
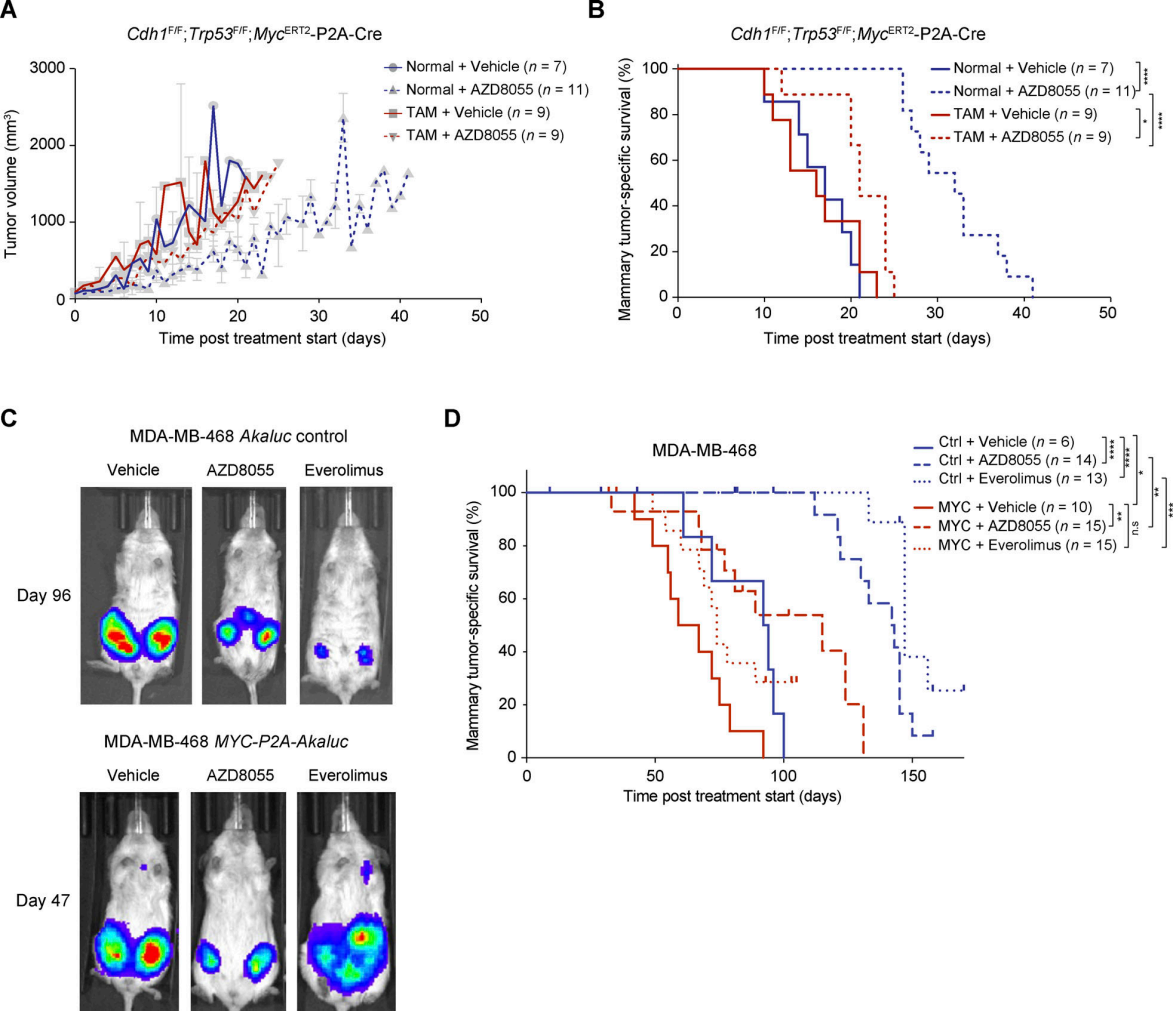
**MYC drives AZD8055 resistance in vivo. (A and B)** Mammary tumor growth curves (A) and Kaplan–Meier curves showing tumor-specific survival (B) of female syngeneic mice bearing mammary fat pad transplants derived from *Cdh1*^F/F^;*Trp53*^F/F^;*Myc*^ERT2^-P2A-Cre tumor donors. Mice were subjected ad libitum to a normal food pellet versus TAM-containing food pellet diet (TAM-induced nuclear translocation of MYC-ERT2) and treated daily orally with vehicle or 20 mg/kg AZD8055. For mammary tumor growth curves in A, data are represented as mean ± SEM of indicated numbers of replica per group. **(C)** Representative in vivo bioluminescence images of immunocompromised female NXG mice intraductally injected with MDA-MB-468 cells transduced with *Akaluc* or *MYC-P2A-Akaluc* lentiviral vectors and treated daily orally with 20 mg/kg AZD8055, 5 mg/kg everolimus, or either of the corresponding vehicles. Akaluciferase activity is shown following Akalumine-HCl administration 96 (*Akaluc*) or 47 (*MYC-P2A-Akaluc*) d after mTORi treatment start. **(D)** Kaplan–Meier curves showing mammary tumor–specific survival of female immunocompromised mice intraductally injected with MDA-MB-468 cells expressing *Akaluc* or *MYC-P2A-Akaluc* and treated daily with vehicles or indicated mTORi. In B and D, P values were calculated with log rank (Mantel–Cox) test (*P < 0.05; **P < 0.01; ***P < 0.001; ****P < 0.0001; n.s, not significant). *n* represents the number of mice per cohort included in the analysis. Mice treated with AZD8055-vehicle or everolimus-vehicle were grouped into single vehicle cohorts.

Our in vitro findings prompted us to address whether MYC was also a tumor cell–intrinsic driver of mTORi resistance in vivo. Firstly, we generated a somatically engineered mouse model allowing us to induce MYC activity in situ in established ILC tumors. To this end, we somatically delivered lentiviruses containing *Myc*^ERT2^-*P2A-Cre* to mammary glands of *Cdh1*^F/F^;*Trp53*^F/F^ (EP) female mice by using intraductal injections (Annunziato et al., 2016; Zingg et al., 2022). This led to the formation of EP-*Myc*^ERT2^ mammary tumors that express an MYC-ERT2 fusion protein, which translocates to the nucleus upon tamoxifen (TAM) binding. Histological examination confirmed emerging tumors to express MYC and to be solid ILCs, consistent with the predominant tumor type emerging from mammary glands of *K14-Cre*;*Cdh1*^F/F^;*Trp53*^F/F^ female mice (Derksen et al., 2006; Klarenbeek et al., 2020; Doornebal et al., 2013). We orthotopically transplanted EP-*Myc*^ERT2^ tumors into the mammary fat pads of immunocompetent female mice. When allografts became palpable, recipient mice were allocated to either normal or TAM-containing food pellets to activate nuclear translocation of MYC-ERT2, which is a well-established method to promote nuclear MYC activity in vivo (Zimmerli et al., 2022). Simultaneously, mice were subjected to daily vehicle versus AZD8055 treatments by oral gavage. AZD8055-mediated mTOR blockade efficiently delayed tumor growth resulting in prolonged tumor-specific survival (Fig. 6, A and B), in agreement with the observed tumoristasis in AZD8055-treated KEP tumors (Klarenbeek et al., 2020). Strikingly, TAM-mediated MYC-ERT2 activation strongly accelerated the growth of EP-*Myc*^ERT2^ tumors treated with AZD8055, resulting in a shortened latency of tumor-specific survival comparable with vehicle-treated controls (Fig. 6, A and B). Second, we orthotopically transplanted human MDA-MB-468 breast cancer cells expressing either *Akaluc* or *MYC-P2A-Akaluc* into the mammary glands of immunocompromised NOD-*Prkdc*^*scid*^-*IL2rg*^*Tm1*^/Rj (NXG) female mice via intraductal injection. Intraductal injection of patient-derived breast cancer cells represents a methodology that well-preserves breast cancer–specific pathology and tumor growth kinetics, and that comprehends superior tumor-take rates over fat pad or subcutaneous transplantations (Valdez et al., 2011; Hutten et al., 2023). When the mammary glands displayed palpable tumors, mice were allocated to vehicle, AZD8055, or everolimus treatment arms. Daily administration of AZD8055 or everolimus via oral gavage suppressed tumor growth, as evidently shown by bioluminescence using the luciferin analog Akalumine (Fig. 6 C), and resulted in significantly prolonged tumor-specific survival of mTORi-treated mice as compared to animals treated with vehicle (Fig. 6 D). Importantly, *MYC* overexpression strongly reduced the efficacy of AZD8055 and everolimus, resulting in shortened mammary tumor–specific survival compared with the mTORi-treated cohort harboring MDA-MB-468 without *MYC* overexpression (Fig. 6, C and D). Collectively, our data demonstrated that MYC drives resistance to clinically approved mTOR-targeted therapies in mouse models of murine and human breast cancer.

### MYC counteracts mTORi-mediated translation inhibition

**Figure 7.**
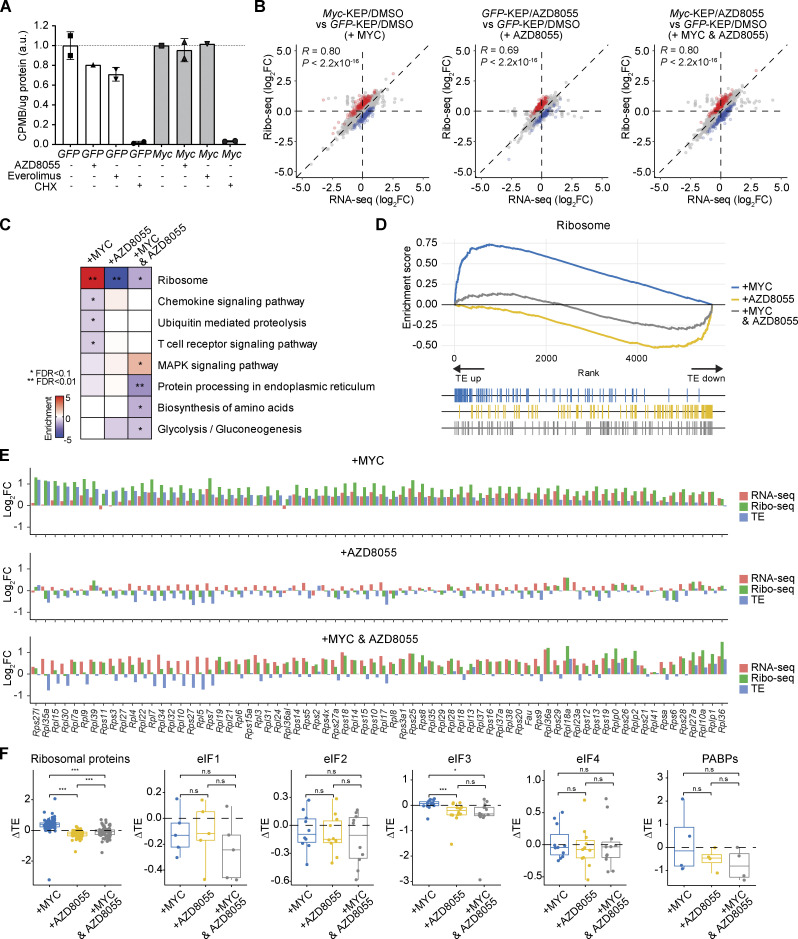
**MYC enhances the translation efficiency of ribosomal genes. (A)** [^35^S]-methionine incorporation assay performed with KEP1.23 cells overexpressing *GFP *or *Myc *and treated for 2 h with vehicle, 50 nM AZD8055, or 10 nM everolimus prior to a 1 h pulse with [^35^S]-labeled methionine. 100 μg/ml cycloheximide (CHX) was added as a positive control to stop protein translation. Incorporation of [^35^S]-methionine was quantified by scintillation counting and normalized to total protein. Data are represented as mean ± standard deviation of *n* = 2 technical replica per group (*GFP*-DMSO, *GFP*-everolimus, *GFP*-CHX, *Myc*-AZD8055, and *Myc*-CHX) or *n* = 1 replica (*GFP*-AZD8055, *Myc*-DMSO, and *Myc*-everolimus) of one experiment and normalized to their corresponding DMSO treated condition. **(B)** Correlations of log_2_(FC) values between Ribo-seq reads and RNA-seq reads in indicated comparisons. Blue and red dots indicate genes showing enhanced or suppressed TEs, respectively. **(C)** Functional enrichment analysis using Kyoto Encyclopedia of Genes and Genomes pathways for genes with significantly altered TEs upon *Myc *overexpression, AZD8055 treatment, or the both, each compared to *GFP*-DMSO. Fisher’s exact test was performed followed by FDR correction. *FDR < 0.05, **FDR < 0.01. **(D)** Gene set enrichment plot showing changes in TEs upon *Myc *overexpression, AZD8055 treatment, or the both, each compared to *GFP*-DMSO. **(E)** Log_2_FC values for all quantified ribosomal genes computed from RNA-seq reads (red), Ribo-seq reads (green), and TE (blue) for each comparison. **(F)** Log_2_FC values of TE (ΔTE) in each condition when compared to *GFP*-DMSO for different protein synthesis factors. Data are represented as median ± IQR (box) and quartiles ± 1.5 × IQR (whiskers), and one-way ANOVA and Tukey’s post-hoc test were performed to compute adjusted P values (*P < 0.05; ***P < 0.001; n.s, not significant).

**Figure S5.**
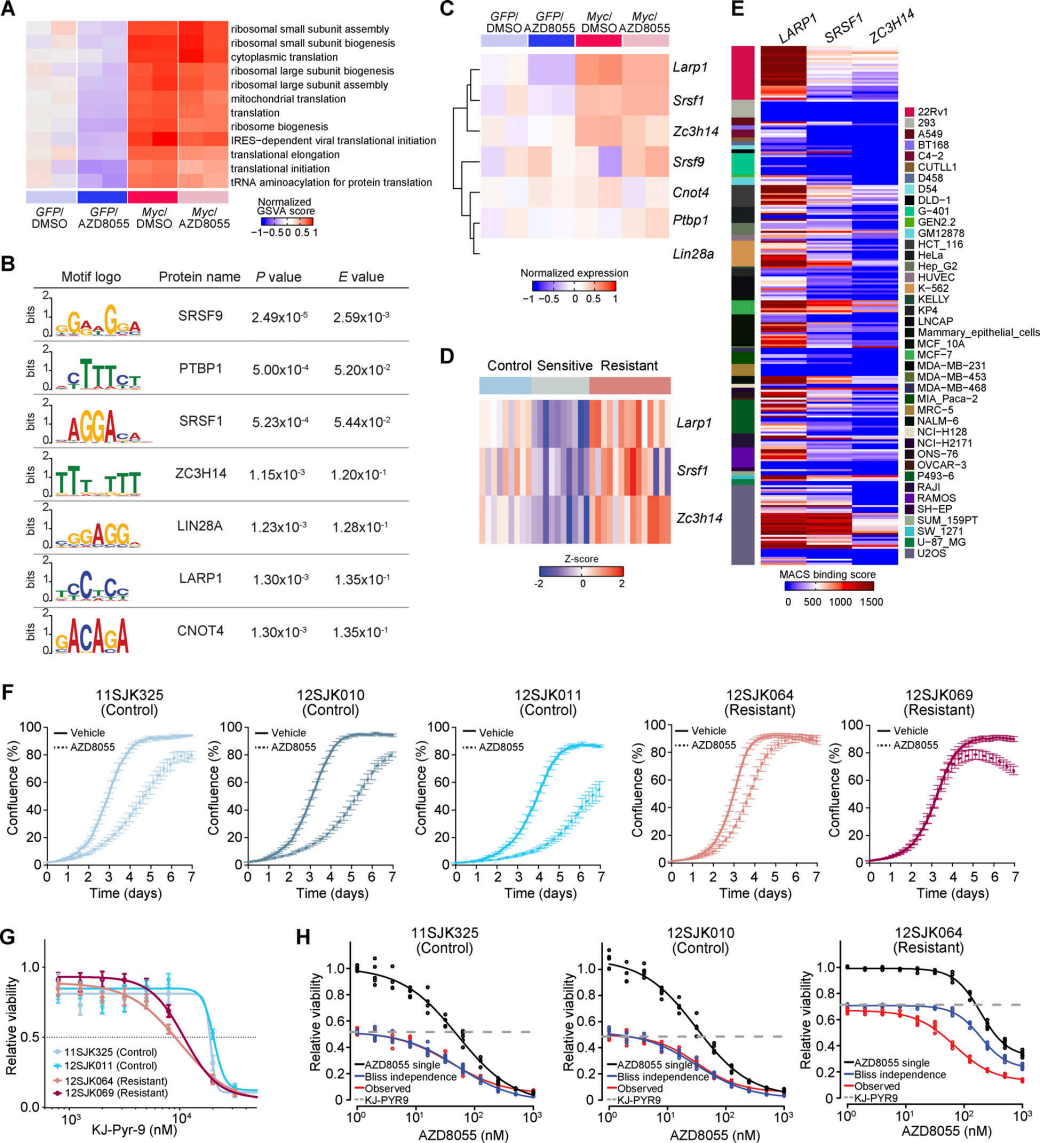
**RNA-binding proteins associated with MYC-driven translation regulation. (A)** Normalized GSVA scores (centered on the mean of control *GFP*/DMSO samples) for translation and ribosome-associated processes in GOBPs computed based on the RNA-seq profiles of *GFP* and *Myc*-overexpressing KEP1.23 cells after DMSO or AZD8055 treatment (two replicas per condition). **(B)** Motifs of RNA-binding proteins that were significantly enriched in 5′-UTRs of transcripts in *Myc*-overexpressing KEP1.23 cells versus controls, as identified using the AME motif scanner (McLeay and Bailey, 2010). Motif logos that were P value (Wilcoxon rank-sum test) adjusted by Bonferroni correction and *E*-value (adjusted P value multiplied by the number of motifs) were provided by AME. **(C)** Gene expression of RNA-binding proteins identified in A in *GFP* and *Myc*-overexpressing KEP1.23 cells after DMSO or AZD8055 treatment (two replicas per condition). Log_2_(CPM) values were normalized by subtracting the mean values of *GFP*-DMSO samples. **(D)** Gene expression of the RNA-binding factors *Larp1*, *Srsf1*, and *Zc3h14* across 9 control, 10 sensitive, and 14 resistant KEP tumors. Log_2_(CPM) values were transformed to Z-scores. **(E)** Model-based analysis of ChIP-seq (MACS) binding scores (−10og_10_Q-value) in the promoter regions (±5KB from transcription start site) of *LARP1*, *SRSF1*, and *ZC3H14* obtained from the ChIP-Atlas database (Oki et al., 2018). **(F)** Incucyte Live-Cell proliferation assay of control and resistant KEP cultures treated with vehicle or 90 nM AZD8055 for 7 d. Data are represented as mean ± SEM of five replicas per group of two independent experiments. **(G)** Dose–response curves of control and resistant KEP cultures, treated with KJ-Pyr-9 for 3 d, and measured by SRB colorimetric assay. Data are represented as mean ± standard deviation of five replicas per group of two independent experiments. **(H)** Dose–response curves of control and resistant KEP cultures treated with an AZD8055 range and 8 μM KJ-Pyr-9 MYCi for 3 d, and measured by SRB colorimetric assay. Bliss independence model is shown to display an independent effect of KJ-Pyr-9 and AZD8055. Data are represented as mean ± standard deviation of five replicas per group of one experiment.

Previous work showed that inhibition of mTOR suppresses protein translation (Jefferies et al., 1997; Yang et al., 2022; Thoreen et al., 2012; Philippe et al., 2020). Consistently, we found that mTOR blockade in KEP tumors suppressed transcriptional programs related to protein translation initiation (Fig. 3, G and H; and Fig. S2 E). In contrast, *Myc* copy number gains in AZD8055-resistant KEP tumors correlated with the upregulation of genes involved in protein translation, as revealed by gene set analysis of RNA-seq data (Fig. 3, G and H; and Fig. S2 E). We functionally addressed whether MYC directly counteracted mTORi-dependent ablation of protein translation. We first measured global translation in control and *Myc*-overexpressing KEP1.23 cells using a [^35^S]-methionine incorporation assay. Both AZD8055 and everolimus treatment suppressed protein translation by 20–30%, whereas global translation in *Myc*-overexpressing cells was unimpaired by mTORi (Fig. 7 A). In line with this observation, evaluation of RNA-seq data derived from KEP1.23 cells showed that AZD8055 treatment resulted in the downregulation of genes related to translation and ribosome processes, whereas *Myc* overexpression strongly induced these processes and considerably counteracted the effect of mTOR blockade (Fig. S5 A).

To elucidate the underlying mechanism of MYC-mediated translation regulation, we performed an integrative analysis of genome-wide ribosome footprints using ribosome profiling (Ribo-seq) and transcriptomes using RNA-seq. This allowed us to calculate translation efficiencies (TEs) of expressed genes and to identify genes with increased or decreased TEs across two experimental conditions, that is, the changes in ribosome footprints were higher or lower as compared with the changes in gene expression between the two conditions. We obtained gene sets that showed TE changes in KEP1.23 cells treated with AZD8055, overexpressing *Myc*, or both versus treatment-naïve control KEP cells (Fig. 7 B). Blockade of mTOR strongly suppressed TEs of genes related to the ribosome (Fig. 7 C). In contrast, overexpression of MYC-induced TEs of ribosomal genes, which significantly mitigated the effect of mTORi on ribosomal gene TEs (Fig. 7, C and D). Most ribosomal gene transcripts showed a stark decrease in ribosome occupancy in AZD8055-treated versus control cells, and *Myc* overexpression counteracted this phenotype by promoting the translation of ribosomal genes (Fig. 7 E). TEs of some ribosomal genes that are highly suppressed by AZD8055 were not restored by MYC overexpression. In line with this observation, we found a significant correlation in TE changes induced by AZD8055 between control KEP cells and *Myc*-overexpressing KEP cells (Pearson’s *R* = 0.7, P = 7.7 × 10^−12^). Of note, MYC did not affect TEs of genes encoding translation machinery factors other than ribosomal proteins (Fig. 7 F). This suggested that MYC overcomes mTORi-mediated translation inhibition by recovering the translation of ribosomal gene transcripts.

To further dissect the connection of MYC to translational regulation, we performed motif enrichment analysis for RNA-binding proteins among the genes that showed increased TEs during *Myc* overexpression (Fig. 7 E). The 5′-UTR regions of these gene transcripts were enriched for motifs of known RNA-binding proteins including LARP1, SRSF1, and ZCRH14 (Fig. S5 B). Interestingly, *Larp1*, *Srsf1*, and *Zc3h14* transcripts were significantly upregulated in KEP cells overexpressing *Myc* as well as in AZD8055-resistant tumors containing focal *Myc* amplifications (Fig. S5, C and D), and the three genes were potential MYC transcriptional targets according to the ChIP Atlas database (Oki et al., 2018; Fig. S5 E). Taken together, these data implied that MYC can exert mTORi resistance through the transcriptional induction of RNA-binding proteins, which in turn fosters the translation of ribosomal proteins to restore protein synthesis during mTOR blockade.

### AZD8055-resistant tumors depend on MYC activity

**Figure 8.**
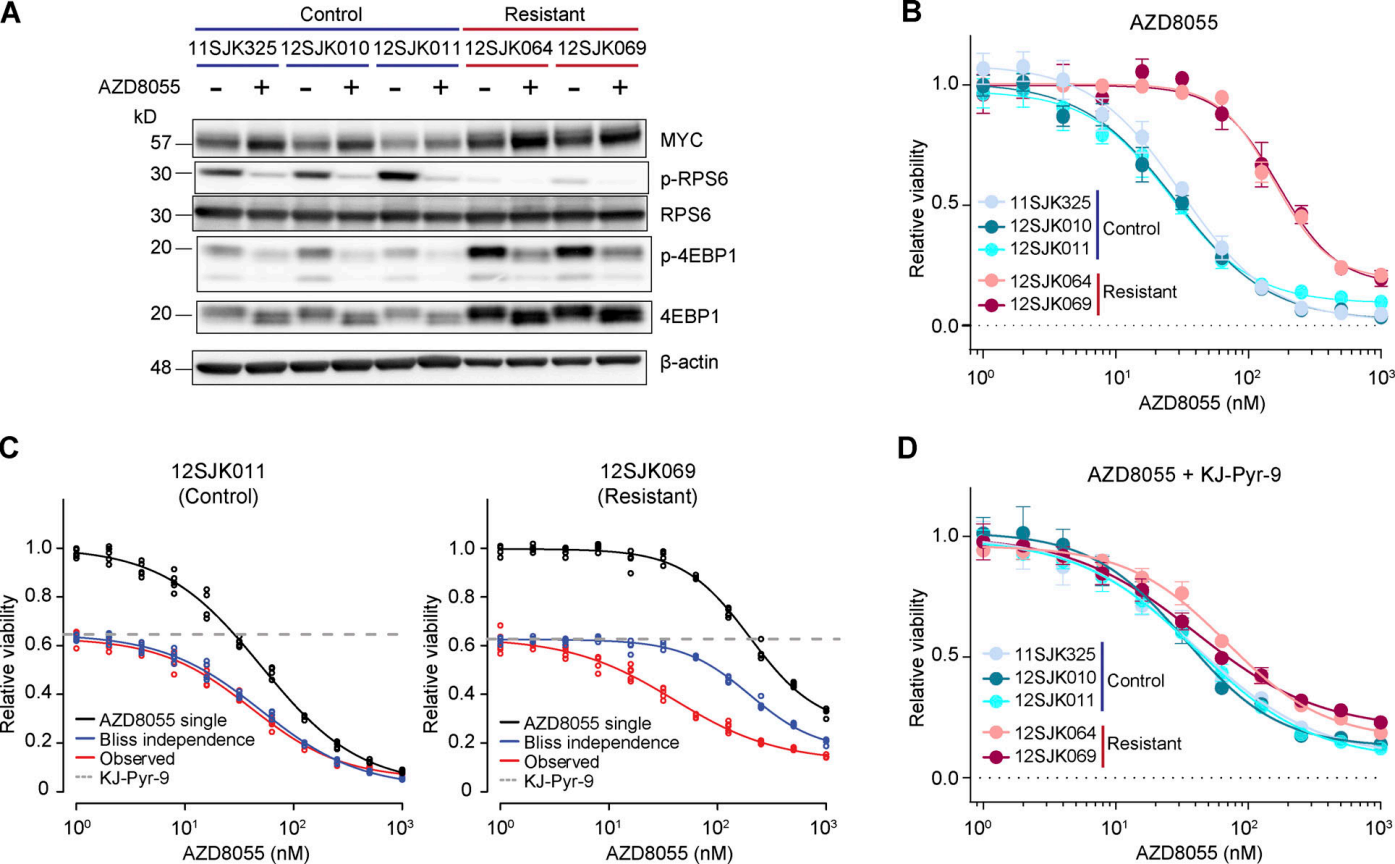
**Synergistic effect of mTOR and MYC inhibition in AZD8055-resistant cell cultures. (A)** Western blots for MYC and mTOR signaling markers on control and AZD8055-resistant, *Myc*-amplified KEP tumor–derived primary cell cultures treated with vehicle, or 90 nM AZD8055 for 24 h. Data represent one experiment of three independent experiments. **(B)** Dose–response curves of control and resistant KEP cultures, treated with AZD8055 for 3 d, and measured by SRB colorimetric assay. Data are represented as mean ± standard deviation of five replicas per group of five independent experiments. **(C)** Dose–response curves of control (12SJK011) and resistant KEP (12SJK069) cultures, treated with an AZD8055 range and 8 μM KJ-Pyr-9 MYCi for 3 d, and measured by SRB colorimetric assay. Bliss independence model is shown to display the independent effect of KJ-Pyr-9 and AZD8055. The dotted line represents the basal effect of 8 μM KJ-Pyr-9 and the solid lines represents dose response curves for AZD8055 single treatment (black), Bliss independence (blue), and observed effect of combination treatment (red). Data are represented as mean ± standard deviation of five replicas per group of one experiment. **(D)** Dose–response curves of control and resistant KEP cultures, treated with 8 uM of MYCi KJ-Pyr-9 in addition to a range of AZD8055 concentrations for 3 d, and measured by SRB colorimetric assay. Data are represented as mean ± standard deviation of five replicas per group of one experiment. Source data are available for this figure: SourceData F8.

Next, we functionally dissected whether the acquired resistance to AZD8055 in KEP tumors depended on the observed *Myc* amplifications. To this end, we established primary cell cultures from KEP tumors that had simultaneously been used for the multiomics analyses. We grew cells from three vehicle-treated control tumors and from two AZD8055-resistant tumors both harboring focal *Myc* amplifications. Cells from control KEP tumors were sensitive to AZD8055 (Fig. 8, A and B). Cells from AZD8055-resistant KEP tumors displayed target inhibition (Fig. 8 A) and maintained resistance to AZD8055 in vitro, as evidenced by an average sixfold IC50 increase in AZD8055-resistant cells versus sensitive control cells (Fig. 8 B) as well as the maintained proliferation of resistant cells during treatment with AZD8055 (Fig. S5 F). We subjected the AZD8055-sensitive and -resistant cell cultures to drug–response assays using the MYC inhibitor (MYCi) KJ-Pyr-9 (Hart et al., 2014). The cell cultures derived from AZD8055-resistant and *Myc*-amplified KEP tumors were more vulnerable to KJ-Pyr-9 as compared with the control cultures (Fig. S5 G). We next assessed whether MYC blockade would resensitize the mTORi-resistant and *Myc*-amplified cells to AZD8055 inhibition. Dose-responses to AZD8055 were measured in the absence or presence of 8 μM KJ-Pyr-9, a dose that roughly corresponded to the KJ-Pyr-9 IC40 observed for AZD8055-resistant cell cultures (Fig. S5 G). MYC inhibition reduced baseline cell viability of both control and AZD8055-resistant cells. Using Bliss independence modeling, which assumes independent actions of two combined drugs (Greco et al., 1995), we predicted the additive effect when combining AZD8055 and KJ-Pyr-9 (“Bliss independence” in Fig. 8 C and Fig. S5 H). Comparison of the observed effect of combination treatment with the Bliss independence prediction showed that MYC blockade had no effect on AZD8055 sensitivity of control KEP cells (5 nM of average ΔIC50 between Bliss model prediction and observed response; Fig. 8 C and Fig. S5 H). In contrast, KJ-Pyr-9 treatment synergistically resensitized mTORi-resistant KEP cells to AZD8055 inhibition (average ΔIC50 of 127 nM; Fig. 8 C and Fig. S5 H). Consequently, upon MYC blockade, the AZD8055 IC50 curves of *Myc*-amplified resistant KEP cultures decreased to a sensitivity level comparable with control KEP cultures (Fig. 8, B and D). Together, these data showed that MYC can confer resistance to AZD8055, and MYC blockade might represent a therapeutic strategy to overcome acquired resistance to mTORi.

### MYC status is associated with poor response to mTORi-based therapy

**Figure 9.**
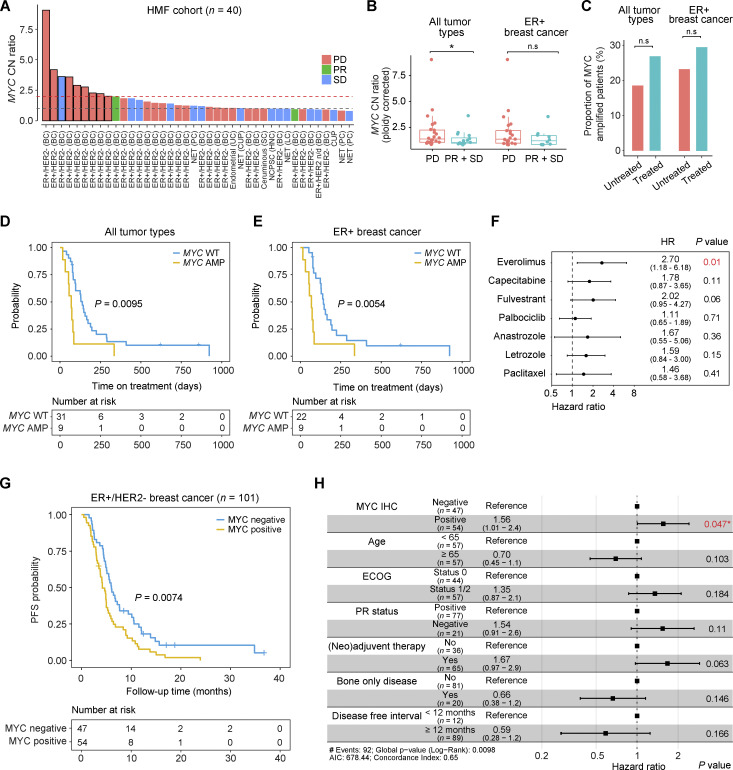
**MYC status is a poor prognostic factor for everolimus-based therapy. (A)** *MYC* CN status of tumors from the HMF cohort (*n* = 40) prior to mTORi treatment. *MYC* CN status was normalized to sample ploidy (SP), and samples with *MYC* CN > 2*SP were defined as *MYC*-amplified tumors (Priestley et al., 2019). Bar plots show *MYC* CN status of samples with clinical response to mTORi treatment. PD, progressive disease. Red dotted line denotes *MYC* amplification cutoff. The gray dotted line denotes normal *MYC* CN status (CN ratio = 1). BC, breast cancer; PC, pancreatic cancer; UC, uterine cancer; SC, skin cancer; HNC, head and neck cancer; LC, lung cancer; NET, neuroendocrine tumor; NCPSC, nasal cavity and paranasal sinus cancer; CUP, cancer of unknown primary; n/d, not determined. **(B)** Comparisons of *MYC* CN status of responders (PR + SD) vs. non-responders (PD) in all tumor types (total, *n* = 40; PR + SD, *n* = 16; PD, *n* = 24) and in ER+ breast cancer (total, *n* = 31; PR + SD, *n* = 9; PD, *n* = 22), specifically. Data are represented as median ± IQR (box) and quartiles ± 1.5 × IQR (whiskers) and Wilcoxon rank-sum test was performed to compute P values (*P < 0.05; n.s, not significant; all tumor types, P = 0.04; ER+ breast cancer, P = 0.13). **(C)** Percentages of tumors with *MYC* amplifications in the pre and post mTORi-treated HMF cohorts (all tumor types, *n* = 121; untreated, *n* = 54; treated, *n* = 67; ER+ breast cancer, *n* = 104; untreated, *n* = 43; treated, *n* = 61). Proportion test was performed to compare the percentages between groups (n.s, not significant; all tumor types, P = 0.19; ER+ breast cancer, 0.31). **(D and E)** Kaplan–Meier curves showing everolimus treatment duration for patients with *MYC* WT or *MYC*-amplified cancers (D; *n* = 40) or specifically ER+ breast cancers (E; *n* = 31) using data from the HMF cohort. P values were calculated with log-rank (Mantel–Cox) test. **(F)** Univariate cox regression analysis to evaluate the association between individual therapeutics used in ER+/HER2− breast cancer and treatment duration from HMF cohort. Data are represented as estimated hazard ratio (HR) ± 95% confidence interval (CI). **(G)** Kaplan–Meier curve showing progression-free survival (PFS) of ER+/HER2− breast cancer patients (*n* = 101) from the everolimus biomarker study (EudraCT number 2013-004120-11) during everolimus/exemestane treatment stratified for MYC positivity using IHC. P values were calculated with log rank (Mantel–Cox) test. **(H)** Multivariate Cox regression analysis on breast cancer specimens from G using indicated clinicopathological variables. For each variable, data are represented as estimated hazard ratio ± 95% CI. ECOG, Eastern Cooperative Oncology Group performance status; PR, progesterone receptor status of the primary tumor of study participants.

To examine the clinical relevance of our findings, we evaluated the association between *MYC* amplification status and mTORi response in tumor samples derived from patients with cancer. We first analyzed WGS data with associated clinical data from the metastatic cancer cohort of the Hartwig Medical Foundation (HMF; Priestley et al., 2019). This collection contained data for 40 cancer patients who received the mTORi everolimus as a single agent or in combination with other drugs. Analysis of genomic profiles from pretreatment biopsies revealed higher *MYC* copy number levels in non-responders (progressive disease) as compared with responders (SD or PR, as defined by response evaluation criteria in solid tumors [RECIST] criteria; Fig. 9, A and B). When focusing on ER+ breast cancer only (30 ER+/HER2− [human epidermal growth factor receptor 2], 1 ER+/HER2 unknown), we also observed a trend toward *MYC* amplification enrichment in non-responding patients (Fig. 9 B). We also observed a trend toward a higher prevalence of *MYC* amplification in the post-mTORi-treatment cohort versus the pretreatment cohort, although the difference did not reach statistical significance (Fig. 9 C). This suggested that *MYC* amplification-driven resistance to mTOR-targeted therapies may also occur in human cancer patients. Because of incomplete survival information for patients receiving everolimus, we used treatment duration as a proxy for progression-free survival (Bögemann et al., 2020; Hari et al., 2018). Patients with *MYC*-amplified tumors received everolimus for a significantly shorter period of time, indicating that everolimus was less effective in these patients (Fig. 9, D and E). Of note, univariate Cox-regression analysis of ER+/HER2− breast cancer samples from the HMF data showed that *MYC* amplification is significantly associated with everolimus-based treatment duration (HR = 2.70, P = 0.01; Fig. 9 F).

Finally, we analyzed 101 ER+/HER2− metastatic breast cancer patients who participated in the everolimus biomarker study (EudraCT number 2013-004120-11) and received everolimus plus exemestane as a standard of care treatment (Kruger et al., 2020). Primary tumor tissues were stained for MYC by IHC, and the percentage of stained tumor cells was quantified to group patients into MYC-positive and MYC-negative samples. The MYC-positive group displayed significantly shortened progression-free survival as compared with the MYC-negative group (Fig. 9 G). Importantly, a multivariate Cox proportional hazard model including clinicopathological characteristics confirmed that, among the variants tested, MYC positivity was the only determinant significantly associated with worse progression-free survival (HR = 1.56, P = 0.047; Fig. 9 H).

Together, these results suggested that MYC is a clinically significant driver of mTORi resistance and that MYC status may potentially stratify patients that may benefit from mTOR-targeted therapy.

## Discussion

The PI3K–AKT–mTOR signaling pathway is frequently activated in breast cancer and has therefore been considered an attractive drug target (Bahrami et al., 2018). However, lack of knowledge about the factors determining response to drugs targeting PI3K–AKT–mTOR signaling including mTORi often hampers the optimal choice of treatment (Lee et al., 2015; Yi and Ma, 2017). In this study, we demonstrated MYC activation to be a central mechanism of mTORi resistance in breast cancer. Resistance to mTORi has previously been investigated in several cancer types, and MYC has been reported as a resistance mechanism (Allen-Petersen et al., 2019; Ilic et al., 2011; Matsumoto et al., 2015; Muellner et al., 2011; Muranen et al., 2016; Shepherd et al., 2013; Song et al., 2021; Tan et al., 2013; Dey et al., 2014). These studies were mostly based on in vitro cell culture experiments (Ilic et al., 2011; Matsumoto et al., 2015; Tan et al., 2013; Allen-Petersen et al., 2019). To recapitulate mTORi resistance acquisition occurring in breast cancer patients in a more appropriate way, we analyzed mammary tumors isolated from immunocompetent mice that were enrolled into a long-term mTORi intervention, we made use of clinically approved mTOR-targeted therapies, and we engaged in mouse modeling using tumor samples of mouse and human origin to functionally pinpoint MYC as an in vivo driver of mTORi resistance. Notably, we also made use of clinical data, which highlighted the clinical potential of MYC to predict mTORi response and to stratify patients that are less likely to benefit from mTORi therapy.

Although MYC was the most prominent hallmark of AZD8055-resistant tumors, copy number analysis also revealed a focal amplification of Chr-2 in three resistant tumors (Fig. 1 D) that was mutually exclusive to the *Myc* amplification. The Chr-2 region covered 35 protein-coding genes, two of which encoded the translation initiation factors EIF3M and EIF5 (Fig. S1 G). We demonstrated that MYC drives mTORi resistance by restoring protein synthesis during mTOR blockade. Conceivably, genes encoded by the Chr-2 amplicon including *Eif3m* and *Eif5* that foster protein translation might thus represent alternative resistance factors to overcome mTOR blockade beyond MYC.

We identified MYC as an acquired resistance factor during mTOR inhibition. Correspondingly, our analyses of human cancer cell line and patient data demonstrated that *MYC* amplification and high *MYC* expression prior to mTORi treatment are associated with poor mTORi response. This suggests that MYC-driven tumors are likely intrinsically resistant to mTOR blockade. Across human cancer, *MYC* is frequently amplified and MYC can mitigate antitumor effects of many cancer therapeutics (Fallah et al., 2017). This stresses the need to identify the Achilles’ heel(s) of MYC-driven cancers. Targeting MYC itself has remained challenging, partly because of the lack of an enzymatic pocket and the predominant nuclear localization of the protein, both of which make the use of small molecule inhibitors or blocking antibodies cumbersome (Wang et al., 2021). However, recent cancer dependency map studies utilizing CRISPR/Cas9 and small hairpin RNA–based genetic screens may represent valuable resources to identify novel therapeutic opportunities to target MYC-dependent cancer cells (Tsherniak et al., 2017; Meyers et al., 2017).

At present, targeted therapies of the PI3K–AKT–mTOR pathway are not first-line in breast cancer. Instead, these therapeutics have been used in ER+/HER2− postmenopausal breast cancer patients who are refractory to endocrine therapies—acquired genetic alterations activating the PI3K–AKT–mTOR pathway frequently drive resistance to endocrine therapies (Nunnery and Mayer, 2020). Our study suggests that MYC drives primary resistance to mTORi and might thus serve as a predictive biomarker for response to second-line PI3K–AKT–mTOR-targeted therapies. Our analysis of the HMF cohort indeed revealed that 32% (10/31) of ER+/HER2− breast cancer patients showed an objective response to the everolimus–exemestane combo-treatment regime (Fig. 9 A). It will be interesting to retrospectively evaluate MYC status using clinicogenomic data from the BOLERO-2 trial or other clinical trials that evaluated everolimus-based therapies for ER+/HER2− breast cancer in different first-line settings (Rozenblit et al., 2021; Dhakal et al., 2018). Considering the potential adversary role of MYC activity in the efficacy of a diverse set of cancer therapeutics, MYC status should also be evaluated in the context of other anticancer therapies. To conclude, our findings suggest that MYC is an important mediator of mTORi response and its status should be considered to select patients who may benefit from mTOR-targeted therapies.

## Materials and methods

### Lentiviral vectors and virus production

The SIN.LV.SF-T2A-Puro, SIN.LV.SF-GFP-T2A-Puro, and SIN.LV.SF-P2A-Cre lentiviral vectors were previously described (Zingg et al., 2022). Mouse *Eif4b* (NM_145625.3), mouse *Melk* (NM_010790.2), and human *MYC* (NM_001354870.1) cDNAs were isolated from custom-synthesized gBlocks Gene Fragments (Integrated DNA Technologies). The mouse *Myc* (NM_010849.4) and *Myc*^ERT2^ cDNAs were isolated from the *Myc* cDNA clone 8861953 (Source BioScience) and the *Frt-invCAG-Myc*^ERT2^*-IRES-Luc* vector (Zimmerli et al., 2022), respectively. The *Akaluc* cDNA was isolated from a previously described vector (Iwano et al., 2018) by using BamHI and AgeI restriction enzymes (#R0136, #R3552; New England BioLabs). cDNAs were amplified using Q5 High-Fidelity DNA Polymerase (#M0491; NEB) and inserted into the SIN.LV.SF-T2A-Puro and SIN.LV.SF-P2A-Cre vectors, resulting in SIN.LV.SF-Eif4b-T2A-Puro, SIN.LV.SF-Melk-T2A-Puro, SIN.LV.SF-Myc-T2A-Puro, and SIN.LV.SF- Myc^ERT2^-P2A-Cre. Human *MYC* together with the P2A linker sequence and *Akaluc* were amplified and assembled into the SIN.LV.SF-T2A-Puro backbone by using the In-Fusion HD Cloning Plus kit (#638911; Takara Bio) according to the manufacturer’s recommendations, resulting in SIN.LV.SF-Akaluc-T2A-Puro and SIN.LV.SF-MYC-P2A-Akaluc-T2A-Puro vectors. Point mutations in *Eif4b* and *Melk* were introduced using the QuikChange Lightning Site-Directed Mutagenesis Kit (#210519; Agilent) and primers designed with QuikChange Primer Design (Agilent). All constructs were verified by Sanger sequencing. Lentiviral stocks were produced by transient co-transfection of four plasmids in HEK 293T cells as previously described (Follenzi et al., 2000). Viral titers were determined using the qPCR Lentivirus Titer Kit (#LV900; Applied Biological Materials).

### Cell culturing and lentiviral transduction

HEK 293T cells (#CRL-3216; ATCC) were cultured in Iscove’s Modified Dulbecco’s Medium (#31980030; Thermo Fisher Scientific) containing 10% fetal bovine serum (FBS; #S-FBS-EU-015; Serana) and 100 IU/ml penicillin and streptomycin (Pen Strep, #15070; Thermo Fisher Scientific). MDA-MB-468 (#HTB-132; ATCC), MCF7 (#HTB-22; ATCC), T47D (#HTB-133; ATCC), and SUM52PE (#HUMANSUM-0003018; BioIVT) cells were cultured in Dulbecco’s Modified Eagle Medium/Nutrient Mixture F-12 (DMEM/F-12, #10565018; Thermo Fisher Scientific) containing 10% FBS, 100 IU/ml Pen Strep, and 10 µM Y-27632 dihydrochloride (#M1817; AbMole). Rac-11P cells (Delwel et al., 1993) were cultured in DMEM medium (#31966-021; Thermo Fisher Scientific) supplemented with 10% FBS and 100 IU/ml Pen Strep. No reauthentication of the abovementioned cell lines was carried out for this study beyond the reauthentications done by the providers. Mouse tumor-derived primary cells as well as the two previously established KEP1.23 and KEP2.E3 cell lines (Klarenbeek et al., 2020) were cultured in DMEM/F-12 containing 10% FBS, 100 IU/ml Pen Strep, 5 ng/ml epidermal growth factor (#E4127; Sigma-Aldrich), 5 ng/ml insulin (#I0516; Sigma-Aldrich), and 10 µM Y-27632 dihydrochloride. All cell lines were cultured in standard incubators at 37°C with 5% CO_2_ and routinely tested for mycoplasma contamination using the MycoAlert Mycoplasma Detection Kit (#LT07-218; Lonza).

Mouse KEP1.23 and KEP2.E3 cell lines were transduced with lentiviral supernatants at equal multiplicity of infections (MOIs). MDA-MB-468, SUM52PE, MCF7, and T47D human breast cancer cell lines were transduced with lentiviral supernatants using a 1–50 MOI range, and resulting experimental control and *MYC*-expressing cell line pairs were selected based on equal RNA expression levels of the corresponding transgenes, as determined by reverse transcription quantitative PCR. Cell transductions were performed in the presence of 8 μg/ml Polybrene (#H9268; Sigma-Aldrich) for 24 h. Transduced cells were selected with 2 μg/ml puromycin (#A11138; Thermo Fisher Scientific) for at least 5 d, and subsequently cultured with 1 µg/ml puromycin to ensure continued transgene expression.

### Isolation of primary tumor cells

50–100 mm^3^ of cryopreserved mammary tumor pieces were thawed on ice and minced using scalpel blades. Minced tumor samples were washed twice with DPBS (#14190144; Thermo Fisher Scientific), digested with 2 mg/ml collagenase type IV (#17104019; Thermo Fisher Scientific), and 4 µg/ml DNase I (#DN25, Sigma-Aldrich) in DMEM/F-12 for 1 h at 37°C, and the cell suspension was passed through a 70-µm cell strainer. Initially, cells were cultured in complete medium supplemented with 5 µM Nutlin-3a (#SML0580; Sigma-Aldrich) to select *Trp53*-deficient cells, which was verified by genotyping PCR as previously described (Derksen et al., 2006). Cells derived from AZD8055-resistant KEP tumors were continuously cultured with 90 nM AZD8055 to maintain the resistance phenotype.

### Cell proliferation assay

400 KEP1.23 or KEP2.E3 cells or 500 primary control or resistant tumor cells were seeded per well in 96-well flat-bottomed plates. After 24 h, cells were subjected to 25 nM AZD8055 (KEP1.23 and KEP2.E3) or 90 nM AZD8055 (primary control and resistant tumor cultures) treatment. Real-time proliferation of the cells was monitored by the Incucyte ZOOM Live-Cell Analysis System (Essen BioScience) over 7 d, and the IncuCyteTM ZOOM 2015A Control software was used to visualize and analyze the results.

### Drug–response assays

400 KEP1.23 or KEP2.E3 cells expressing *GFP* or *Myc*; 1,000 KEP2.E3 cells expressing *GFP*, *Eif4b*, or *Melk*; 700 primary control or resistant tumor cells; 4,000 MDA-MB-468 cells; or 5,000 SUM52PE, MCF7, or T47D cells were seeded per well in 96-well flat-bottomed plates. After 24 h, cells were subjected to either the mTORi AZD8055 (0.1 nM–1 µM range; AstraZeneca) or everolimus (#HY-10218; MedChem Express; 0.03–32 nM range), the PI3Kα inhibitor alpelisib (#HY-15244; MedChem Express; 80 nM–80 µM range), the pan-class I PI3Ki buparlisib (#HY-70063; MedChem Express; 10 nM–10 µM range), or the MYCi KJ-Pyr-9 (#HY-19735; MedChemExpress; 800 nM–80 µM range), each for 3 d. To measure cell viability upon combined mTOR and MYC inhibition, cells subjected to AZD8055 were additionally treated with 8 μM KJ-Pyr-9. Cell viability was assayed using Sulforhodamine B (SRB) staining. Cells were fixed with 50% ice-cold trichloroacetic acid in H_2_O for 2 h at 4°C, washed five times with tap water, and air-dried. Cells were then stained for 30 min with 0.4% SRB–1% acetic acid in H_2_O at room temperature and washed five times with 1% acetic acid-H_2_O. Bound SRB was dissolved with 10 mM Tris-H_2_O and optical density was measured at 540 nm with an Infinite 200 PRO plate reader (Tecan). For KEP2.E3 cells expressing *Eif4b* or *Melk*, cell viability was assayed using CellTiter-Blue reagent (#G808A; Promega) for 4.5 h and an Infinite M Plex plate reader (Tecan). Long-term colony formation assays were performed in 6-well plates precoated with laminin by using Rac-11P cells as previously described (Schipper et al., 2019). Cells were seeded at 5,000 cells per well and treated the following day at the indicated concentrations. After 10 d of treatment, cell viability was quantified by incubating the cells with Cell-Titer Blue reagent. Cells were then fixed with 4% formaldehyde and stained with 0.1% (wt/vol) crystal violet. Plates were imaged using GelCount (Oxford Optronix). Drug–response curves were modeled using [Inhibitor] versus response with variable slope (four parameters) and least squares regression in Prism (version 8, GraphPad Software). The Bliss independence model was applied to evaluate synergy between AZD8055 and KJ-Pyr-9 (Greco et al., 1995).

### CRISPR/Cas9-mediated genome editing

For CRISPR/Cas9-mediated genome editing of *Mtor* (NM_020009.2), *Rictor* (NM_030168.3), and *Rptor* (NM_028898.2), two independent single-guide (sg)RNAs per gene were selected from the Brie Mouse CRISPR Knockout Pooled Library (Doench et al., 2016). Sequences of non-targeting and *Mtor*-, *Rptor*-, and *Rictor*-targeting sgRNAs were as follows: non-targeting, 5′-TGA​TTG​GGG​GTC​GTT​CGC​CA-3′; *Mtor*-sg1, 5′-TGC​AGT​CTG​GCT​AAC​CAC​GT-3′; *Mtor*-sg2, 5′-GCT​GAT​GCA​CGT​GAA​TAC​GG-3′; *Rptor*-sg1, 5′-GAT​GTG​TCA​AGG​ATT​CGT​TG-3′; *Rptor*-sg2, 5′-TGC​AGG​TCG​TAT​ATG​GAC​AG-3′; *Rictor*-sg1, 5′-TGA​CAT​TCA​GCA​GAG​CAA​CG-3′; *Rictor*-sg2, 5′-TAC​CTG​GAT​CTA​GCA​CGA​TG-3′. sgRNAs were cloned into the lentiCRISPRv2-Cas9-Blast backbone (#98293; Addgene) containing *Cas9* and a blasticidin resistance cassette. DNA oligos were annealed with T4 Polynucleotide Kinase (#M0201; New England BioLabs). The lentiCRISPRv2-Blast backbone was digested with the BsmBI-v2 restriction enzyme (#R0739; New England BioLabs) for 2 h at 55°C, and the correct band was isolated from a 1% agarose gel using the Isolate II PCR and Gel Kit (#BIO-52060; Bioline). Oligos were ligated into the linearized and digested backbone using the Quick Ligation Kit (#M2200; New England BioLabs) for 10 min at 23°C and transformed and expanded in *Stbl3* bacteria. All constructs were verified by Sanger sequencing.

20,000 KEP1.23 GFP or MYC-overexpressing cells were seeded per well into 6-well plates 24 h prior to transfection with lentiCRISPRv2-Cas9-Blast plasmids containing control or sgRNAs of interest using Xfect (#631317; Takara) according to the manufacturer’s instructions. 24 h later, cells were trypsinized and replated with fresh media containing 10 µM blasticidin. After 2 d of blasticidin selection, a cell pellet from the first time point (t1) was collected and frozen, with a split of the cells continuing in culture without blasticidin. Cell pellets were subsequently collected at days 4 (t2) and 6 (t3) of initial blasticidin selection start, respectively. Cell pellets were lysed with DirectPCR Lysis Reagent (#302-C; Viagen), PCR-amplified, and sent for Sanger sequencing using primers designed with SnapGene (version 6.1.2; Dotmatics) and compatible with TIDE analysis (Brinkman et al., 2014). Sequencing results were used to estimate the spectrum and frequency of small insertions and deletions (indels) generated in the different samples and time points following the TIDE analysis methodology (Brinkman et al., 2014).

### Protein isolation and Western blotting

Cells were lysed in radioimmunospecipitation buffer (50 mM Tris-HCl, pH 8, 100 mM NaCl, 1 mM EDTA, 1% NP40, 0.5% sodium deoxycholate, and 0.1% sodium dodecyl sulfate) containing Halt Protease and Phosphatase Inhibitor Cocktail (#78440; Thermo Fisher Scientific). Lysates were cleared by centrifugation at 4°C and protein concentrations were determined with the BCA Protein Assay Kit (#23227; Thermo Fisher Scientific) using an Infinite 200 PRO plate reader. BlueEye Prestained Protein Marker (#PS-104; Jena Bioscience) and 20 µg of protein were separated on NuPAGE 4-12% Bis-Tris Mini Protein Gels (#NP0323; Thermo Fisher Scientific) using NuPAGE MOPS SDS Running Buffer (#NP0001; Thermo Fisher Scientific), and transferred overnight at 4°C onto Nitrocellulose Membranes (#88018; Thermo Fisher Scientific) in transfer buffer (25 mM Tris, 2 M Glycine, 20% methanol in demineralized water). Membranes were stained with Ponceau S (#ab270042; Abcam) and imaged with Fusion FX (Vilber), blocked in 5% bovine serum albumin (BSA; #A8022; Sigma-Aldrich) in PBS-T (0.05% Tween-20), and incubated in 5% BSA in PBS-T overnight at 4°C with the following primary antibodies: β-actin (1:10,000, #A5441; Sigma-Aldrich), C-MYC (1:1,000, #ab32072, Abcam), and phospho (T37/T46)-4E-BP1 (#2855), 4E-BP1 (#9644), phospho (S406)-eIF4B (#8151), phospho (S422)-eIF4B (#3591), eIF4B (#3592), phospho (S235/S236)-S6 ribosomal protein (#2211), and S6 ribosomal protein (all 1:1,000, #2217; all Cell Signaling Technology). Membranes were washed with PBS-T and incubated with anti-rabbit HRP (1:10,000, #P0448; DAKO) or anti-mouse HRP (1:10,000, #G-21040; Thermo Fisher Scientific) secondary antibodies in 5% BSA in PBS-T for 1 h at room temperature. Membranes were then washed in PBS-T, developed using SuperSignal West Pico PLUS Chemiluminescent Substrate (#34580; Thermo Fisher Scientific), and imaged with Fusion FX (Vilber).

### Mouse models

To generate mouse ILC tumors deficient for *Cdh1* and *Trp53* and simultaneously expressing *Myc*^ERT2^, 6-wk-old FVB/NCrl *Cdh1*^F/F^;*Trp53*^F/F^ female mice, genotyped as previously described (Derksen et al., 2006), were intraductally injected with a lentivirus encoding *Myc*^ERT2^-P2A-Cre using a previously established methodology (Annunziato et al., 2016). Briefly, 20 μl of high-titer lentivirus was injected into the third and fourth mammary glands by using a 34G needle. Lentiviral titers ranging from 2 × 10^8^ to 2 × 10^9^ TU/ml were used. Mice were weekly monitored for tumor development and sacrificed when reaching the humane endpoint.

To allograft tumors, cryopreserved 1 mm^3^ tumor fragments derived from the *Cdh1*^F/F^;*Trp53*^F/F^;*Myc*^ERT2^-P2A-Cre (EP-*Myc*^ERT2^) model were orthotopically transplanted into the right mammary fat pad of 8-wk-old syngeneic FVB/NCrl female mice (Janvier labs). Mice were twice weekly weighed and monitored for mammary tumor development and, as soon as tumors reached a volume of 62.5 mm^3^ (5 × 5 mm, measured in two dimensions using a caliper; $volume=length\times {width}^{2}\times 0.5$), mice were randomly allocated to vehicle versus AZD8055 mTORi treatment arms and normal food pellets versus TAM 400-citrate pellets (#TD55125; Envigo) to induce nuclear translocation of MYC-ERT2. The treatments were performed daily via oral gavage using vehicle (0.5% hydroxypropylmethylcellulose and 0.1% TWEEN 80 in demineralized water) or 20 mg/kg AZD8055 while food pellets were provided ad libitum. Mice were sacrificed 1 h after the last AZD8055 dosing when reaching the humane endpoint. AZD8055 intervention studies using cryopreserved KEP ILC pieces were previously described (Klarenbeek et al., 2020) using the same AZD8055 regimen as described above for EP-*Myc*^ERT2^ allografts. Vehicle-treated control tumors were harvested after 5 d or when they reached a volume of 1,500 mm^3^. Sensitive tumors were harvested after 5 d of AZD8055 treatment. Resistant tumors were obtained when mice reached the humane endpoint as stated above.

For the human breast cancer patient-derived xenograft model, 25,000 MDA-MB-468 cells overexpressing *Akaluc* or *MYC-P2A-Akaluc* were injected into the fourth mammary glands of 6-wk-old immunodeficient NXG (Janvier labs) female mice, as previously described (Valdez et al., 2011; Hutten et al., 2023). Animals were weighed and monitored for mammary tumor development twice weekly and, as soon as tumors reached a volume of 62.5 mm^3^ as per the above-mentioned formula, mice were randomly allocated to AZD8055, everolimus, or the corresponding vehicle treatment arms. Mice treated with AZD8055-vehicle or everolimus-vehicle showed no obvious differences in tumor growth kinetics and were thus grouped into single-vehicle control cohorts for MDA-MB-468-*Akaluc* and MDA-MB-468-*MYC-P2A-Akaluc*–bearing mice, respectively. AZD8055 daily treatment regimen was 20 mg/kg AZD8055 in 0.5% hydroxypropylmethylcellulose and 0.1% TWEEN 80 in demineralized water as described for the EP-*Myc*^ERT2^ allografts. Everolimus was administered daily via oral gavage at 5 mg/kg as a microemulsion solution diluted with 5% glucose in demineralized water. Mice were sacrificed 1 h after the last mTORi dosing when reaching the humane endpoint. For a subset of mice within each treatment group, tumor development was additionally monitored once a week by bioluminescence using an IVIS Spectrum instrument (PerkinElmer). Animals were anesthetized prior to the administration of 0.05 mg/kg of Akalumine-HCl substrate (courtesy of Leiden University) diluted in water. The bioluminescence signal was normalized to radiance (photons/second/centimeter^2^/steradian), and the region of interest tool was used to determine the signal of individual tumors. IVIS was stopped after vehicle groups reached the humane endpoint.

For all mouse experiments, endpoint was reached when the total mammary tumor burden was a volume of either 1,500 mm^3^ for a single tumor or 2,000 mm^3^ for cumulative tumors or the mice suffered from clinical signs of distress, such as respiratory distress, ascites, distended abdomen, rapid weight loss, and/or severe anemia, caused by primary tumor burden or metastatic disease. The maximal permitted disease endpoints were not exceeded in any of the experiments. The sample size was determined using G*Power software (version 3.1; Faul et al., 2009) and was large enough to measure the effect size. Tumor measurements and post-mortem analyses were performed in a blinded fashion. The mouse colony was housed in a certified animal facility with a 12-h light/dark cycle in a temperature-controlled room. Mice were kept in individually ventilated cages, and food and water were provided ad libitum. All animal experiments were conducted under the Central Animal Testing Committee licenses number 9 and 24 (CCD 9, AVD3010020172464, appendix 2; CCD 24, AVD30100202011584, appendix 1), approved by the Animal Ethics Committee of the Netherlands Cancer Institute, and performed in accordance with institutional, national, and European guidelines for animal care and use.

### Histology and IHC of mouse tumors

Tissues were formalin-fixed and paraffin-embedded (FFPE) by routine procedures and sectioned for H&E histochemical staining and IHC stainings, which were performed as previously described (Gogola et al., 2018) using primary antibodies against E-cadherin (1:200, #3195; Cell Signaling Technology), C-MYC (1:100, #ab32072; Abcam), MHC-II (1:100, #NBP2-21789; Novusbio), CD4 (1:1,000, #14-9766-82; eBiosience), FOXP3 (1:200, #14-5773; eBioscience), phospho STAT1 (1:800, #9167; Cell Signaling Technology), CD3 (1:600, #RM-9107-S1; Thermo Fisher Scientific), LY6-G (1:150, #551459; BD Pharmingen), B-220 (1:4,000, #557390; BD Pharmigen), CD8 (1:2,000, #14-0808-82; eBioscience), or NKp46 (1:400, #AF2225; R&D Systems). For E-cadherin, C-MYC, MHC-II, CD4, phospho STAT1, CD3, CD8, and NKp46 IHC stainings, antigen retrieval was done with Tris-EDTA at pH 9; for FOXP3 and B-220 IHC stainings, it was done with Citrate Buffer (#CBB999; Scytek); and for LY6-G, it was done with 20 µg/ml Proteinase-K (#P6556; Sigma-Aldrich). Sections were incubated with primary antibodies overnight at 4°C. Primary antibodies against E-cadherin, C-MYC, CD3, and phospho STAT1 were labeled with the EnVision+ HRP Labelled Polymer Anti-Rabbit System (K4003; Dako); primary antibodies against MHC-II, B-220, CD8, LY6-G, and FOXP3 were labeled with Goat-anti-Rat-Bio (1:150, #3052-08; Biotech) and subsequently streptavidin/HRP (1:200, #P0397; Dako); and the primary antibody against NKp46 was labeled with Rabbit-anti-Goat-Bio (1:100, #E0466; Dako) and subsequently streptavidin/HRP. Secondary antibodies were then visualized with the Liquid DAB+ Substrate Chromogen System (#K3468; Dako) and counterstained with hematoxylin. The antibodies used were independently validated by certified pathologists by evaluation of IHC results in positive and negative biological control FFPE tissues to ensure specificity and sensitivity. In addition, negative technical controls for each primary antibody were performed. EP-*Myc*^ERT2^ mammary tumors were classified as solid ILCs using H&E and E-Cadherin slides, and according to the international consensus of mammary pathology (Cardiff et al., 2000). Slides were digitally processed using a PANNORAMIC 1000 whole slide scanner (3DHISTECH), captured with CaseViewer software (version 3.2.2.1, 3DHISTECH), and quantified using HALO Image Analysis Platform (version 3.5.3577; Indica Labs). MYC IHC stains were HALO-quantified by calculating a histo (H)-score for each tumor. H-score was defined as follows: H-score = 1 × (% tumor cells with weak staining intensity) + 2 × (% tumor cells with moderate staining intensity) + 3 × (% tumor cells with strong staining intensity), resulting in a score between 0 and 300. IHC stains for immune markers were HALO-quantified by counting marker-positive cells per 0.25 mm^2^ tumor area to calculate immune cell densities per area. Per tumor, ten 0.25-mm^2^ regions were randomly selected for quantification. IHC stains for MYC and immune markers were performed on consecutive slides and stains quantified using matched regions. All slides and quantifications thereof were reviewed by a comparative pathologist (X. Chao) in a blinded manner.

### LC-WGS data generation and analysis

Genomic DNA from KEP control, sensitive, and resistant tumors was isolated using the ISOLATE II Genomic DNA Kit (#BIO-52066; Bioline) according to the manufacturer’s guidelines. LC-WGS for copy number analysis was performed using double-stranded DNA (dsDNA) and quantified with the Qubit dsDNAHS Assay Kit (#Q32851; Invitrogen). 2 mg of dsDNA were fragmented by Covaris shearing and purified using 1.8× Agencourt AMPure XP PCR Purification beads according to the manufacturer’s protocol (#A63881; Beckman Coulter). Next, sheared DNA was quantified on a BioAnalyzer system with the DNA7500 assay kit (#5067-1506; Agilent Technologies). Library preparation for Illumina sequencing was carried out with 1 mg of DNA and KAPA HTP Library Preparation Kit (#KK8234; KAPA Biosystems). To obtain a sufficient yield for sequencing, four to six PCR cycles were performed during the library enrichment step. Prepared libraries were cleaned up using 1× AMPure XP beads and analyzed on a BioAnalyzer system using the DNA7500 chips to determine the molarity. Finally, up to 11 uniquely indexed samples were pooled (equimolar pooling) in a final concentration of 10 nM and sequenced on an Illumina HiSeq2500 machine in one lane of a single read 65 bp run, according to the manufacturer’s instructions. Sequencing reads were mapped to the human reference genome GRCh38 using Burrows-Wheeler Alignment (BWA-MEM, version 11 0.7.5a; Li and Durbin, 2009). Reads with mapping quality lower than 37 were excluded. The resulting alignments were analyzed with QDNAseq (version 1.14.0) using sequence mappability and guanine-cytosine content correction and a bin size of 20,000 bp to generate segmented copy number values (Scheinin et al., 2014).

### RNA-seq data generation and analysis

Total RNA extraction was performed for frozen tissue samples from KEP control, sensitive, and resistant tumors, as well as for KEP cell lines transduced with lentivirus containing *GFP* or *Myc* and treated with DMSO or 50 nM AZD8055 using the RNeasy Mini Kit (#74104; Qiagen) according to the manufacturer’s guidelines. Quality and quantity of RNA were assessed using the 2100 Bioanalyzer system and a Nano chip (Agilent). RNA samples with RNA integrity number >8 were subjected to polyA-stranded library preparation using the TruSeq RNA Library Prep Kit v2 (#RS-122-2001/2; Illumina) according to the manufacturer’s instructions, quality-checked with the 2100 Bioanalyzer system using a 7,500 chip, and pooled equimolar into a 10-nM sequencing stock solution. Libraries from KEP tumors were sequenced with 100 bp paired-end reads using the HiSeq 2500 System with V4 chemistry and libraries from KEP cell lines were sequenced with 51 bp paired-end reads using NovaSeq 6000. Sequencing reads were mapped to the mouse reference genome Ensembl GRCm38 using STAR (version 2.7.2; Dobin et al., 2013). For gene expression analysis, read counts were quantified by featureCounts (version 1.6.2; Liao et al., 2014). Genes with count per million (CPM) values higher than one in at least 10% of the total number of samples were considered expressed and used for downstream analysis. Read counts for expressed genes were normalized by the Trimmed Mean of M-value method using edgeR (version 3.26.6; Robinson and Oshlack, 2010). Differential expression analysis was performed using limma-voom (version 3.52.4; Ritchie et al., 2015). For TE analysis, read counts were quantified by HTSeq (version 1.99.2; Anders et al., 2015) for the transcript that represents each gene. The representative transcript for a gene was defined as the one that shares most exons with other transcripts in the gene, using CGAT (version 0.6.5; Sims et al., 2014).

### Ribo-seq data generation and analysis

1.4 million GFP or MYC-overexpressing KEP1.23 cells were seeded in 10-cm dishes and after 24 h subjected to DMSO or 50 nM AZD8055 treatment, which represented an IC80 for GFP cells and an IC30 for MYC cells. Ribosome-protected fragments sequencing libraries were constructed as previously described (Ingolia et al., 2012). In brief, 24 h after treatment, cells were pretreated with cycloheximide (100 μg/ml) for 1 min, followed by detergent lysis and ribosome footprinting by RNase I digestion. Library constructions were generated from 26 to 34 nt footprint fragments and sequenced with 75-bp single-reads using the NextSeq HIGH system (Illumina). Sequencing reads were first trimmed to remove adaptor sequences in reads using the Cutadapt (version 3.5; Martin, 2011) and resulting reads shorter than 20 bp were discarded. Reads derived from rRNAs and tRNAs were filtered by alignment to sets of mouse rRNA and tRNA references using bowtie2 (version 2.4.5; Langmead and Salzberg, 2012). The remaining reads were aligned to the mouse reference genome Mouse_M15_CTAT_lib_Nov012017 using the Tophat2 (version 2.1.1; Kim et al., 2013). Only primary alignments with mapping qualities of 10 or greater were used for the quantification of a representative transcript for each gene using HTSeq (version 1.99.2; Anders et al., 2015). The transcript that shared most exons with other transcripts in the gene was selected as a representative transcript using CGAT (version 0.6.5; Sims et al., 2014).

### Analysis of TE and RNA-binding motif

TE was calculated by dividing the number of ribosome footprint reads by the number of mRNA reads. DESeq2 (version 1.30.1) was used to normalize RNA-seq and Ribo-seq read counts for representative transcripts and subsequently calculate TE based on a design formula that took into account the sample groups (GFP-vehicle, GFP-AZD8055, MYC-vehicle, MYC-AZD8055) and the data type (RNA-seq, Ribo-seq): counts ∼ group + group*data type. The interaction terms were used to compute TEs in each group, and the changes in TE were calculated by comparing the coefficient of the interaction term between groups. RNA-binding motif enrichment analysis was performed using the analysis of motif enrichment (AME; version 5.4.1; McLeay and Bailey, 2010) embedded in the MEME Suite (Bailey et al., 2009). 5′-UTR sequences of transcripts that were upregulated by MYC were against previously reported RNA binding motifs (Ray et al., 2013; Hong et al., 2017).

### [^35^S]-methionine incorporation assay

400,000 GFP or MYC-overexpressing KEP1.23 cells were seeded into 6-well plates and after 24 h subjected to media containing DMSO, 50 nM AZD8055, or 10 nM everolimus. Cells were incubated for 2 h at 37°C. For the last 20 min, complete media was substituted by DMEM methionine-free media (#21013024; Thermo Fisher Scientific). Subsequently, cells were incubated with 7 μl [^35^S]-methionine label (#NEG772007MC; PerkinElmer) for 1 h at 37°C. As positive control, 100 μg/ml cycloheximide was added with [^35^S]-methionine to block protein synthesis. Subsequent cell harvesting, cell lysis, and protein precipitation steps were previously described (Silva et al., 2022). Scintillation was measured using a liquid scintillation counter (PerkinElmer) and the activity was normalized by total protein content, as determined by Bradford Assay (#5000006; Bio-Rad).

### MS data generation and analysis

Peptides were labeled with TMT 10plex isobaric tags (Pierce) according to the manufacturer’s protocol, including an internal standard consisting of equal aliquots of all digests to be analyzed. For global proteome analysis, fractions were concatenated to 22 fractions and 11 fractions; for phosphoproteome analysis, fractions were concatenated to five fractions. For phosphoproteomic analysis, phosphorylated peptides were enriched from concatenated fractions using a High-Select Fe-NTA Phosphopeptide Enrichment Kit (Thermo Fisher Scientific), according to the manufacturer’s instructions. Prior to MS data analysis, the peptides used for proteome analysis were reconstituted in 2% formic acid. Peptide mixtures were analyzed by nanoLC-MS/MS on an Orbitrap Fusion Tribrid mass spectrometer equipped with a Proxeon nLC1000 system (Thermo Fisher Scientific; Ameziane et al., 2015). Global proteome data were analyzed by Proteome Discoverer (version 2.2.0.388) and phosphoproteome data were analyzed by MaxQuant (version 1.5.8.3). MS/MS data were searched against the *Mus musculus* Swissprot database (17,397 entries, release 2018_02) using Mascot. False discovery rates (FDRs) for peptide and protein identification were set to 1%, and as additional filter Mascot Ions score >20 was set. The TMT dataset was quantified using Most Confident Centroid with the “reporter ions quantifier” node. For both global and phosphoproteome data, the intensity ratio (log_2_ scale) divided by the intensities of internal control was processed by quantile normalization followed by batch correction using Combat (Leek et al., 2012).

### GSVA

For RNA-seq and MS-based global proteomics data, the GSVA (Hänzelmann et al., 2013) was performed based on MSigDB Hallmark (Liberzon et al., 2015) and GO gene sets (Carbon et al., 2009). Log_2_ scale of CPM for RNA-seq and normalized abundance ratio for global proteomics data were used to perform GSVA with the default parameters (kcdf = “Gaussian,” mx.differ = “TRUE”). Gene sets smaller than 10 were excluded from the GSVA analysis.

### Phosphosite-specific signature analysis

Phosphosites identified from MS-phosphoproteomics analysis of KEP tumors were first mapped to the orthologous phosphosites in human proteins using the PhosphoSitePlus database (https://www.phosphosite.org). The phosphosites without corresponding human orthologous sites were excluded. Then, normalized TMT intensities for filtered phosphosites were averaged for each KEP tumor group to obtain average intensities for control, sensitive, and resistant tumors. Finally, post-translational modification (PTM) signature enrichment analysis was performed based on the PTMSigDB database to identify significantly enriched upstream signals for each tumor group (Krug et al., 2019).

### Analysis of public pharmacological dataset

AUC values for mTORi and copy number status of *MYC* for CCLE human cancer cell lines were obtained from the cBioPortal database (Gao et al., 2013). Data for the mTORi AZD8055, rapamycin, and temsirolimus and the PI3Ki BEZ235, JW-7-52-1, omipalisib, OSI-027, and QL-VIII-58 were examined to associate *MYC* amplification status with PI3Ki/mTORi response. AUC values were compared between cell lines with or without *MYC* amplifications by one-tailed Student’s *t* test. A dataset of 30 breast cancer cell lines including response data for the mTORi AZD8055, BEZ235, MK2206, and GDC0941 was also used to examine *MYC* amplification versus PI3Ki/mTORi response association (Jastrzebski et al., 2018). Copy number estimates (log_2_ ratio) obtained from DNA sequencing were used to define *MYC* amplification (log_2_ ratio >1). log_10_(IC50) values were compared between cell lines with and without *MYC* amplification and *MYC* amplification by one-tailed Student’s *t* test. To measure the correlation between MYC protein and AZD8055 response in human breast cancer cell lines, MYC protein abundances measured by MS were obtained from the Cancer Cell Line Encyclopedia (CCLE) database (Barretina et al., 2012) and AZD8055 response was obtained from the Genomics of Drug Sensitivity in Cancer (GDSC) database (Yang et al., 2013). The HCC70 cell line was excluded from this correlation analysis due to an apparent discrepancy between MS data, which showed very high protein abundance, and the RNA-seq and RPPA data, which indicated low RNA and protein levels, respectively.

### Analysis of gene dependency dataset

Gene dependency data (CRISPR, Public 22Q1) was obtained from the DepMap database (Tsherniak et al., 2017) to evaluate the essentiality of the mTOR complex components MTOR, RPTOR, and RICTOR for cancer cell survival. *MYC* copy number status from Cell Model Passports (van der Meer et al., 2019) was used to compare gene deletion effects in cancer cell lines containing *MYC* amplifications versus lines without *MYC* amplifications.

### Analysis of HMF dataset

Processed WGS data on metastatic tumors before or after receiving everolimus and clinical information were obtained from HMF under data-sharing agreement DR-184. The data can be obtained through standardized procedures and request forms online (https://www.hartwigmedicalfoundation.nl/en/). The WGS data was analyzed based on the HMF bioinformatics pipeline (https://github.com/hartwigmedical/pipeline). In addition, clinical information on those patients was obtained, including treatment response and treatment duration. Treatment response was measured using the RECIST criteria. Copy number values larger than two times the sample ploidy were defined as amplification according to the earlier HMF WGS study (Priestley et al., 2019). The samples with low purity (<35%) and no available treatment response and treatment duration information were excluded from downstream analysis. In total, 40 patients including 31 breast cancer patients were analyzed in this study.

### IHC of MYC in ER+/HER2− breast cancer samples

FFPE blocks from primary tumor of the patients participating in the everolimus biomarker study (EudraCT number 2013-004120-11) were collected as previously described (Kruger et al., 2020). The study was approved by the Independent Ethics Committee of Amsterdam University Medical Centers and Institutional Review Board at each participating site and was performed in compliance with Good Clinical Practices, the Declaration of Helsinki, and carried out in keeping with applicable local law(s) and regulation(s). Also, all patients signed informed consent before enrolment, which included, among others, recording the efficacy of treatment, if possible a non-osseous tumor biopsy at baseline, and the retrieval of archival primary tumor tissue. Tissue microarrays (TMAs) were constructed using three 0.6-mm cores taken from the blocks. Paraffin sections were cut at 3 μm, heated at 75°C for 28 min, and deparaffinized in the instrument with EZ prep solution (Ventana Medical Systems). Heat-induced antigen retrieval was carried out using Cell Conditioning 1 (Ventana Medical Systems) for 64 min at 95°C. IHC staining was done on an automated BenchMark XT Slide Stainer (Ventana Medical Systems) using a C-MYC primary antibody (Y69, 6504612001; Roche Ventana) according to the manufacturer’s protocol. Bound antibody was detected using the OptiView DAB Detection Kit (Ventana Medical Systems). Slides were counterstained with Hematoxylin II and Bluing Reagent (Ventana Medical Systems). The stained TMA slides were scanned and the percentage of positive tumor cells (0–100%) was scored using Slide Score (https://www.slidescore.com). For log-rank test and multivariate Cox-regression analysis, patients were divided into MYC-positive (>0%) and MYC-negative (0%) groups depending on the presence of MYC-positive tumor cells on the TMA slides. Details on the collection of clinicopathological information from ER+/HER2− metastatic breast cancer patients treated with everolimus and exemestane were previously described (Kruger et al., 2020).

### Online supplemental material

The supplementary information provides an overview of multiomic molecular profiling of KEP tumors performed in this study (Fig. S1), differences in biological processes represented by different KEP tumor groups (Fig. S2), associations between MYC status and mTOR complex dependency, as well as the response to PI3K and mTOR inhibitors (Fig. S3), in vitro drug response assay for inhibitors of PI3K and mTOR in human breast cancer cell lines (Fig. S4), and RNA-binding proteins associated with MYC-driven translation regulation (Fig. S5).

## Supplementary Material

SourceData F1 is the source file for Fig. 1.

SourceData F4 is the source file for Fig. 4.

SourceData F5 is the source file for Fig. 5.

SourceData F8 is the source file for Fig. 8.

## Data Availability

The KEP tumor sequencing and MS proteomics data underlying Figs. 1, 2, 3, S1, S2, and S5 are available in the European Nucleotide Archive under the accession number PRJEB46418 and Proteomics Identification Database under the accession number PXD041927, respectively. RNA- and Ribo-seq data for KEP cell lines underlying Figs. 7 and S5 are available in the European Nucleotide Archive under the accession number PRJEB61759. CCLE, GDSC, and cBioPortal pharmacogenomic data underlying Fig. 4 are available through the respective data portals (Barretina et al., 2012; Yang et al., 2013; Gao et al., 2013). Gene dependency data underlying Fig. S3 are available in the DepMap portal (https://depmap.org/portal/). WGS and clinical metadata underlying Fig. 9 were provided by the HMF under license number DR-185 and can be obtained through standardized data request procedures (https://www.hartwigmedicalfoundation.nl/en).
