# Supplementary figures and images for: MYC is a clinically significant driver of mTOR inhibitor resistance in breast cancer
Source: J Exp Med. 2023 Aug 29;220(11):e20211743. doi: 10.1084/jem.20211743 (PMC10465700; doi:10.1084/jem.20211743)

Source Data Supplementary Figure 1. (E)

E.

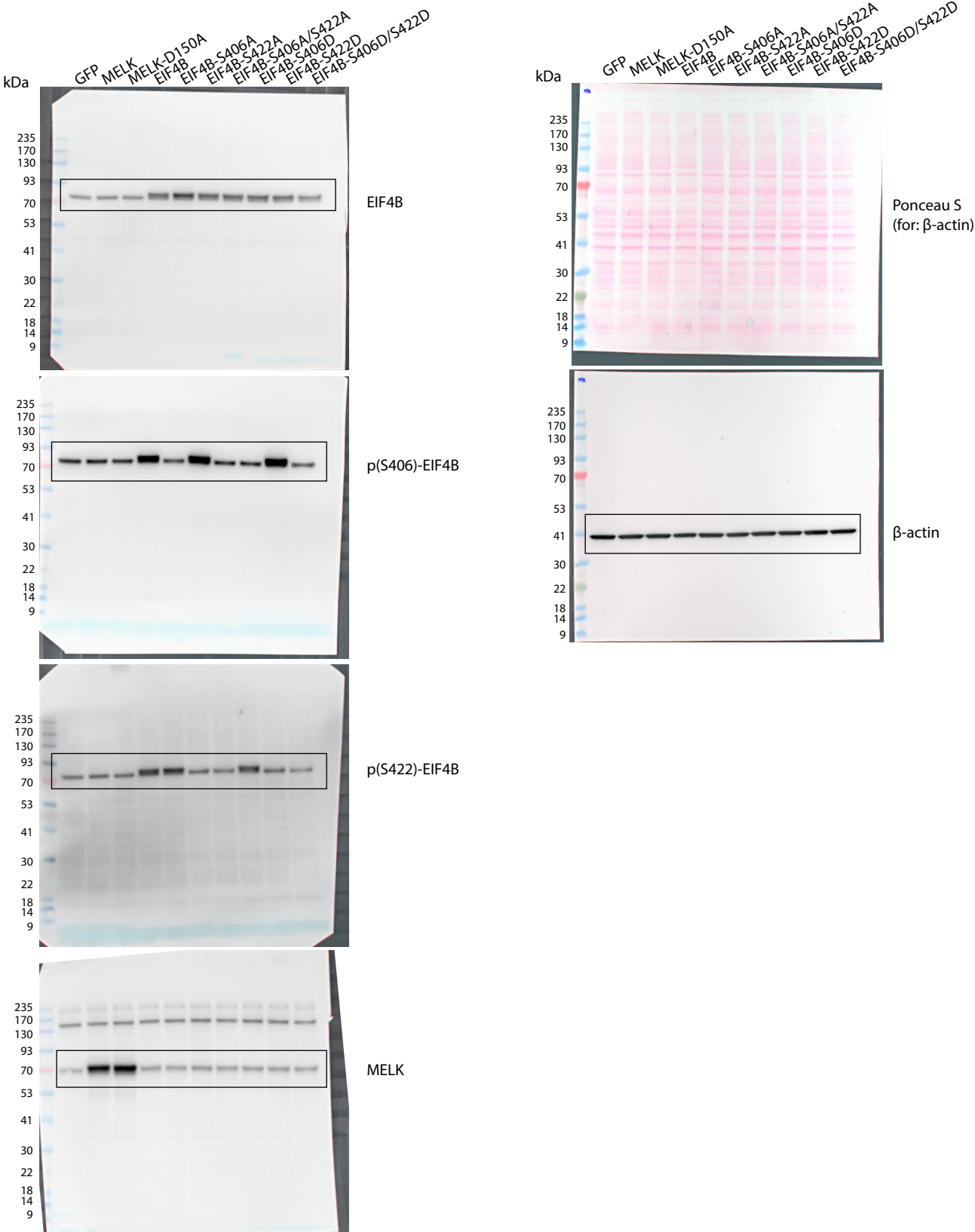

Supplement: SourceData F1 — is the source file for Fig. 1. [file JEM_20211743_SourceDataF1.pdf]

Source Data Supplementary Figure 4. (A)

A.

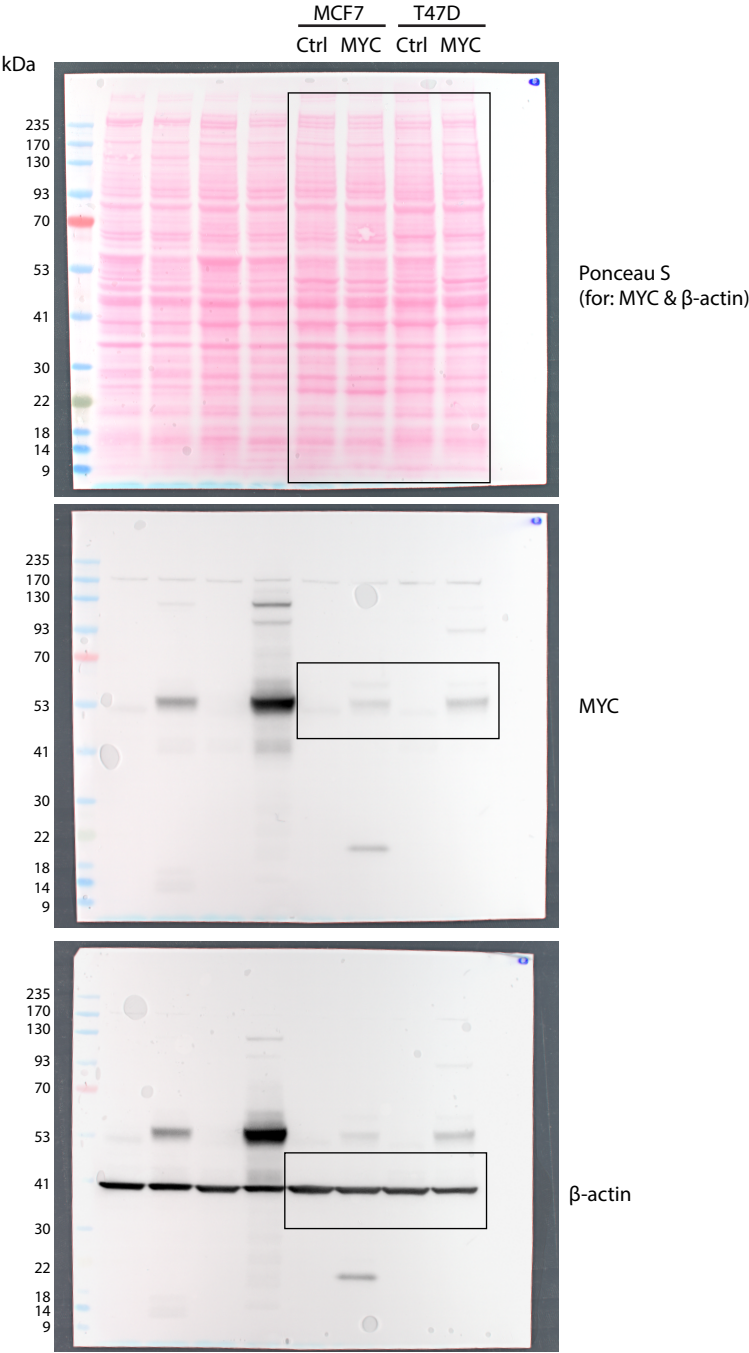

Supplement: SourceData F4 — is the source file for Fig. 4. [file JEM_20211743_SourceDataF4.pdf]

Source Data Figure 5. (A) and (D)

A.

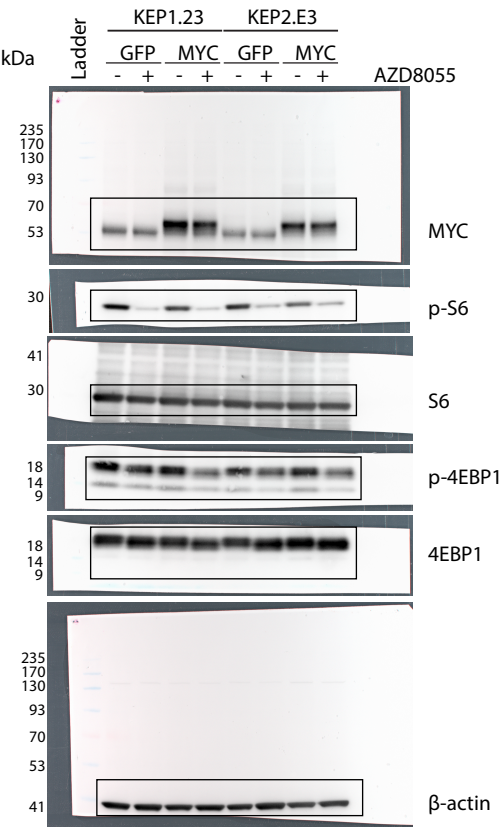

D.

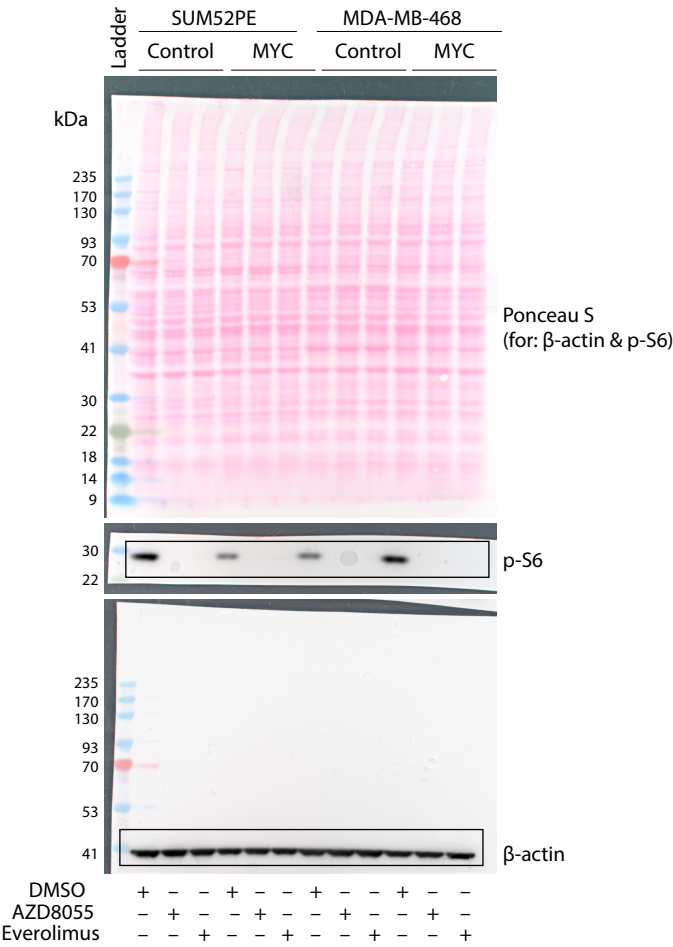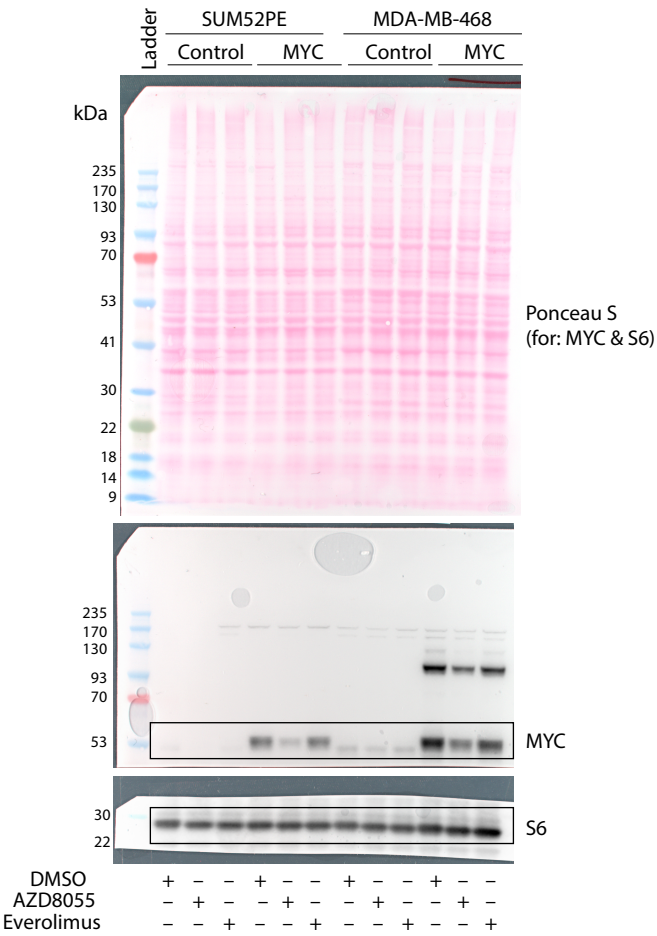

Supplement: SourceData F5 — is the source file for Fig. 5. [file JEM_20211743_SourceDataF5.pdf]

Source Data Figure 8. (A)

A.

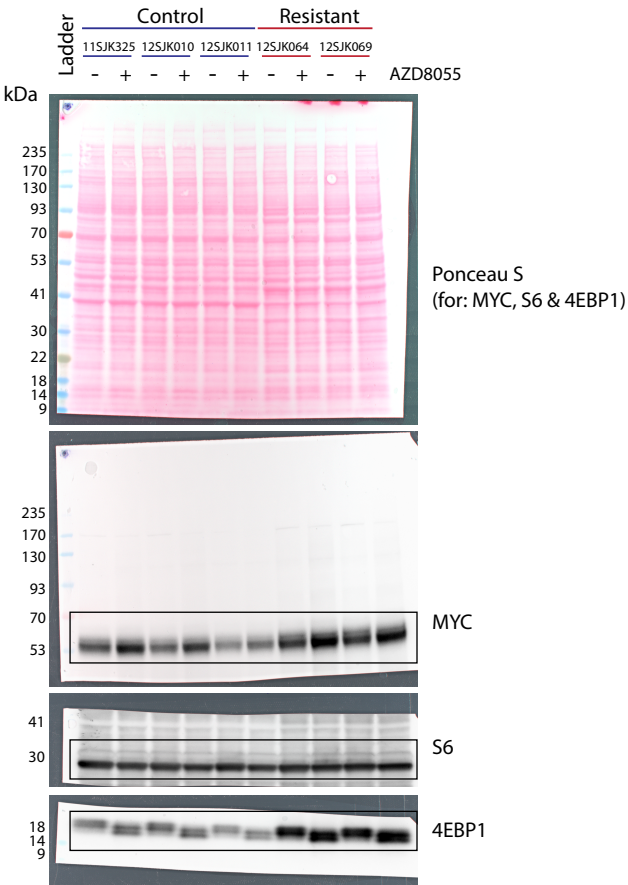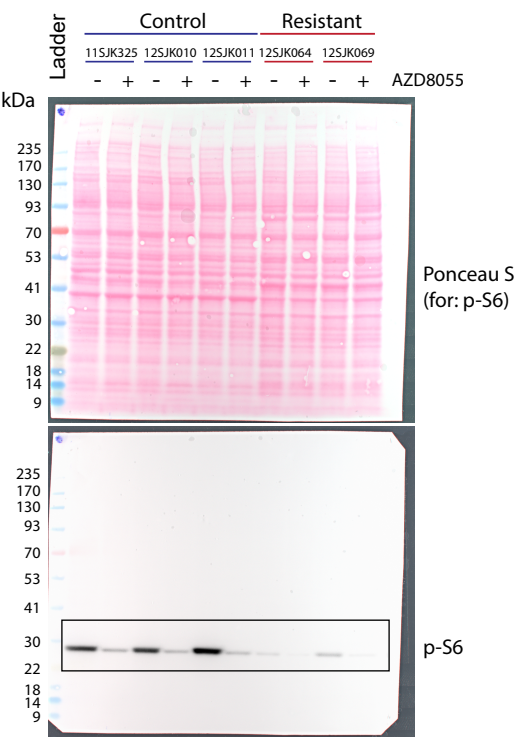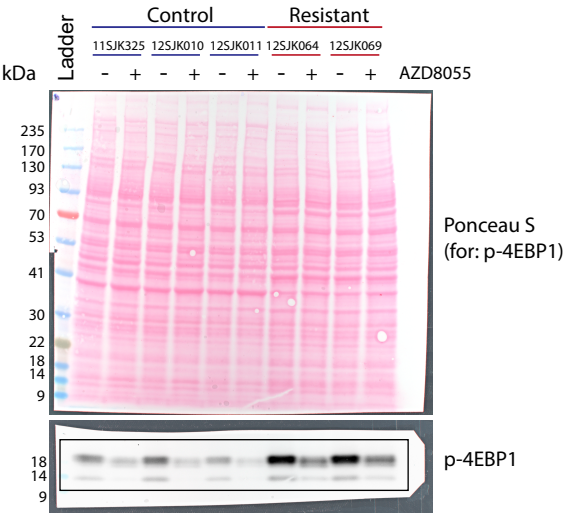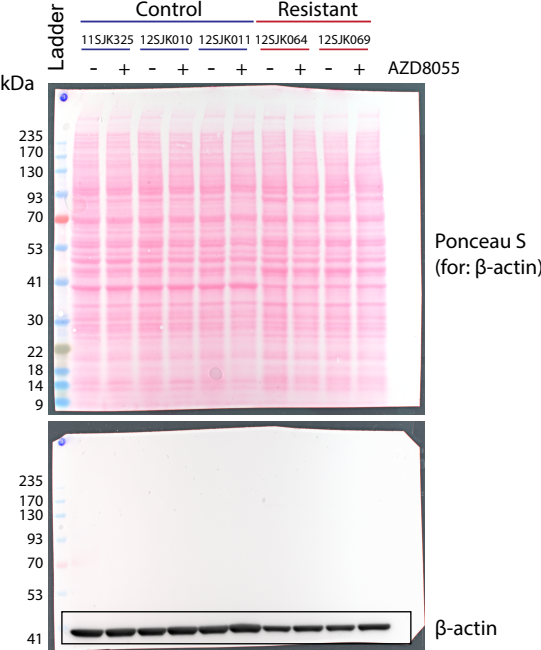

Supplement: SourceData F8 — is the source file for Fig. 8. [file JEM_20211743_SourceDataF8.pdf]
